# Quantifying differences in water and carbon cycling between paddy and rainfed rice (*Oryza sativa* L.) by flux partitioning

**DOI:** 10.1371/journal.pone.0195238

**Published:** 2018-04-06

**Authors:** Bhone Nay‐Htoon, Wei Xue, Steve Lindner, Matthias Cuntz, Jonghan Ko, John Tenhunen, Christiane Werner, Maren Dubbert

**Affiliations:** 1 LIFT / UNOPS Technical Assistant Team, Department of Rural Development, Ministry of Agriculture, Livestock and Irrigation, Nay Pyi Taw, Myanmar; 2 Department of Plant Ecology, University of Bayreuth, Germany; 3 Tree- and Ecosystem-level Integrative Ecology and Ecophysiology, INRA, Nantes, France; 4 Department of Applied Plant Science, Chonnam National University, Gwangju, South Korea; 5 Department of Ecosystem Physiology, University of Freiburg, Freiburg, Germany; Universidade Federal de Santa Maria, BRAZIL

## Abstract

Agricultural crops play an important role in the global carbon and water cycle. Global climate change scenarios predict enhanced water scarcity and altered precipitation pattern in many parts of the world. Hence, a mechanistic understanding of water fluxes, productivity and water use efficiency of cultivated crops is of major importance, i.e. to adapt management practices. We compared water and carbon fluxes of paddy and rainfed rice by canopy scale gas exchange measurements, crop growth, daily evapotranspiration, transpiration and carbon flux modeling. Throughout a monsoon rice growing season, soil evaporation in paddy rice contributed strongly to evapotranspiration (96.6% to 43.3% from initial growth to fully developed canopy and amounted to 57.9% of total water losses over the growing seasons. Evaporation of rainfed rice was significantly lower (by 65% on average) particularly before canopy closure. Water use efficiency (WUE) was significantly higher in rainfed rice both from an agronomic (WUE_*agro*_, i.e. grain yield per evapotranspiration) and ecosystem (WUE_*eco*_, i.e. gross primary production per evapotranspiration) perspective. However, our results also show that higher WUE in rainfed rice comes at the expense of higher respiration losses compared to paddy rice (26% higher on average). Hence, suggestions on water management depend on the regional water availability (i.e. Mediterranean vs. Monsoon climate) and the balance between higher respiratory losses versus a potential reduction in CH_4_ and other greenhouse gas emissions. Our results suggest that a shift from rainfed/unsaturated soil to waterlogged paddy conditions after closure of the rice canopy might be a good compromise towards a sustainable use of water while preserving grain yield, particularly for water-limited production areas.

## Introduction

Rice (*Oryza Sativa* L.) is an important food source for half of the current world’s population and the global demand for rice is projected to increase along with increasing global population [1, 2]. More than 80% of global rice production area is located in Asia [3, 4] and 80% of it is cultivated under conventional flooded conditions [5, 6]. Rice grown under flooded conditions consumes 1000 to 5000 l of water to produce 1 kg of grain and is also reported for its high methane (CH_4_) emission [3, 7]. Therefore, several water saving rice production techniques were introduced, which also aim at mitigating CH_4_ emission [8, 9]. On the other hand, decreased crop yields under water limited conditions are reported [10], although it is a crop which can be grown under different water regimes [11].

Agricultural land-use-changes such as shifting conventional flooded paddy rice to dry-land rice farming further impacts carbon and water exchange of rice ecosystems [3, 12]. Even in conventional paddy rice systems, intensity and timing of flooding and drainage regulation influences the seasonal carbon and water balance [13–15]. Although previous studies report differences in ecosystem carbon and water balance of paddy and rainfed rice [15, 16], a detailed quantification of the contribution and seasonal dynamics of the productive (i.e. gross primary productivity and transpiration) and unproductive components (respiration and evaporation) of ecosystem carbon and water exchange is still lacking.

We studied carbon and water exchange of paddy (conventional flooded system) and rainfed rice (non-irrigated) of the same rice variety over a whole growing season by combining classical chamber flux measurements, high resolution remote sensing by Unmanned Aerial Vehicle (UAV) and crop growth modeling.

The overarching goal of this study was to quantify the impact of water management practice (i.e. a conversion from paddy to rainfed conditions) on the carbon and water balance of an entire growing season. Specifically, we wanted to test whether the expected lower water loss under unsaturated rainfed conditions outweighs the equally expected higher ecosystem respiration compared to water logged conditions. Moreover, the progressive development of the rice canopy throughout the growing season will significantly influence not only the seasonal development of plant water use (transpiration) but also of evaporation from the soil/water surface [17]. Hence, we want to analyze how seasonal changes in the component fluxes of evapotranspiration and net carbon exchange (i.e. transpiration, evaporation, gross carbon uptake and ecosystem respiration) influence cumulative carbon and water budgets as well as water use efficiencies from both ecosystem and agronomic perspectives.

## Materials and methods

### Study site

The study was conducted in the Chonnam National University research farm, (35° 10' N, 126° 53' E, alt. 33m), Gwangju, Chonnam province, Republic of Korea (South Korea). The Chonnam province is one of the major rice growing regions of South Korea, which has a typical East Asian monsoon climate with an annual mean temperature of 13.8°C (±5.74) and annual mean precipitation of ~1391 mm during the past 30 years (1981–2010). More than 60% of precipitation events occurred during the monsoon season (May to October). Both paddy and rainfed rice fields have similar soil properties with loamy texture and pH 6.5. Detailed soil properties are indicated in Table 1.

**Table 1:** Soil chemical and physical properties of study area, Chonnam National University research farm, Gwangju, S. Korea.

Parameters	Values
pH (1:5)*	6.5 (0.1)
Total organic carbon (Cgkg^-1^)	12.3 (0.5)
Total N (gKg^-1^)	1.0 (0.2)
Available P (mgP_2_O_5_kg^-1^)	13.1(0.7)
CEC (comkg^-1^)	14.4 (0.4)
Texture	Loam (Sand: Silt: Clay = 40: 37: 23)
Field capacity	0.28 m^3^m^-3^

Values were mean values of six replicates and standard errors in Parentheses.

*the ratio of soil: water

Rice (*Oryza sativa* L. subsp. *japonica* cv. *unkwang*) was cultivated as rainfed dryland crop and flooded paddy crop. In both rainfed and paddy rice fields, N: P: K fertilizer (11:5:6) at a rate of N fertilizer (115 kg ha^-1^) was applied as a 80% as basal dosage and 20% during the tillering stage. P fertilizer (62 kg ha^-1^) was applied as a 100% basal dosage. K fertilizer (60 kg ha^-1^) was applied as 65% basal dosage and 35% during tillering. All field management practices of paddy rice and fertilizer dosages reflected the practices of farmers in the region. Under rainfed conditions, rice was directly seeded and no additional irrigation was applied to natural precipitation and no drainage was practiced, although there might be some minimal surface runoff during a heavy rain event. The paddy rice field was additionally irrigated and drainage was applied at the later growth stage. The paddy field had embankments to retain the water, while rainfed rice did not. The experiment was conducted in a randomized complete block design with three replications for each cropping practices (ie., flooded paddy and rainfed dryland). Both fields are side by side (approximately 100m far from each other) while flooded paddy field is 73.0 m x 19.5 m and rainfed rice field is 37.5 m x 28.0 m. Prior to this experiment, rainfed dryland field was cropped with Barley, with zero fertilizer application and minimum tillage. Flooded paddy field was formerly cropped with paddy rice, with fertilizer application of at the same amount with this experiment. Filed management activities by DOY could be seen in S1 Table.

### Environmental variables

Environmental data (global radiation, precipitation, air temperature, relative humidity and wind speed) were continuously collected at 2 m height with an automatic weather station every five minutes (WS-GP1, Delta-T Devices Ltd., UK) and half hourly mean values were logged. Photosynthetic photon flux density (PPFD, LI−190, LI−COR, USA) was measured directly above the crop canopy (~20 cm above the canopy and inside the chamber). Air temperature (T_*air*_) (at ~20 cm above the canopy) inside the carbon and water flux measurement chamber (*see details in the following section*) was also measured by custom-built temperature sensor. Soil temperature at root zone was manually measured along with gas exchange measurements using temperature probes (Conrad, Hirschau, Germany). Soil temperature and volumetric water content (5TE and 10HS, respectively, Decagon, Washington, USA) were measured at 5, 10, 20, 30 and 60 cm depth in each experiment plot. 15 min averaged data from 5TE sensors were stored in a datalogger (Em 50, Decagon, Washington, USA) and 30 min averaged data from 10HS sensors were stored in a datalogger (CR1000, Campbell Scientific, Logan, UT, USA).

On the days of flux measurements, aboveground biomass of plants adjacent to vegetation plots were harvested. Leaf area (LA) was determined with a Leaf Area Meter (LI−3000A, LI−COR, USA) and leaf area index (LAI) was calculated as leaf area per ground area. Total aboveground biomass was collected, dried (60°C, 48 hours) and weighed. Plant height of representative plants was manually measured every month.

### Canopy carbon and water flux measurements

Canopy fluxes were measured on canopy vegetation plots (3 replications per treatment) and soil respiratory fluxes and soil evaporation fluxes were measured on baresoil plots (3 replications per treatment). For both canopy fluxes and baresoil fluxes measurement, soil collars were permanently installed soon after seeding of rainfed and planting of paddy rice. CO_2_ and H_2_O fluxes of rainfed rice were measured by a custom built open chamber constructed according to Pape [18] and successfully tested by Dubbert [19]. H_2_O fluxes were measured by a Cavity Ring-Down Spectrometer (CRDS, Picarro, Santa Clara, USA) and CO_2_ fluxes were measured by a portable Infra-Red Gas Analyzer (LI–820, LI–COR, USA). Both carbon and water fluxes were calculated as differential CO_2_ or H_2_O concentration (i.e. the CO_2_ or H_2_O concentration difference between the air samples taken from the chamber inlet and outlet). Air inlet to the chamber was stabilized by a buffer bottle (200 L). Outlet air from the chamber was pumped to the analyzers via tubes heated up to 38 º C to avoid condensation.

As the heavy weight of the open chamber was hard to handle in paddy soil conditions, CO_2_ fluxes of paddy rice were measured by custom built closed chambers [20, 21]. CO_2_ fluxes from both chambers did not differ significantly (t-test; n.s.). H_2_O fluxes were only measured in rainfed rice since open and flow-through chamber type was more suited to measure H2O fluxes [18, 19]. Ecosystem respiration (R_*eco*_) was measured by insulated opaque PVC dark chambers on crop canopy. Soil respiration (R_*soil*_) was measured from bare soil plots next to the vegetation plots. Data were collected from 6:00 hr to 20:00 hr in one and a half hour interval. Fluxes were recorded within 10 minutes of placing the chambers on soil collar. Diurnal courses of canopy fluxes were recorded during four important crop growth stages, namely; seedling (DOY 140 to 170; one diurnal measurement for each treatment and respective replicates.); tillering (DOY 170 to 180; one diurnal measurement for each treatment and respective replicates.); heading (DOY 200 to 210; two diurnal measurement for each treatment and respective replicates.); maturity (DOY 210 to 220; one diurnal measurement for each treatment and respective replicates.).

Gross Primary Production was calculated as:

$$\mathrm{GPP}=(\mathrm{-NEE})+R_{\mathrm{eco}}$$ (1)

where GPP is gross primary production, NEE is net ecosystem CO_2_-exchange and R_*eco*_ is ecosystem respiration. Total daytime fluxes were calculated by linearly integrating hourly carbon and water fluxes from 6:00 to 20:00 hr.

### UAV remote sensing, modeling daily NDVI

An Unmanned Aerial Vehicle (UAV) equipped with Miniature Multiple Camera Array (Mini MCA) (Tetracam, Inc., USA) with 450, 550, 650, 800, 830, and 880 nm bands and 10 cm ground resolution at 300 m altitude was used. For radiometric calibration of MCA images, calibration targets (black, white and gray) were set up next to the paddy field. A cropscan instrument (Cropscan Inc., USA.) was used to calibrate and evaluate the reflectance data obtained by the UAV system.

Remote sensing images were analyzed by ENVI software (Exelis Visual Information Solutions, Inc., USA.). Three sampling points for each treatment plots of both rainfed and paddy rice were used to calculate normalized difference vegetation index (NDVI) as:

$$\mathrm{NDVI}=\frac{\mathrm{NIR-Red}}{\mathrm{NIR+Red}}$$ (2)

Remote sensing campaigns were carried out at noon of DOY 172, 192, 206, 220 and 233. For daily crop ET and GPP modeling, daily NDVI was modeled by GRAMI crop growth model (S5 Fig; briefly, it simulates daily crop growth based on growing degree day, radiation use, daily carbohydrate production by crop canopy, conversion from carbohydrate to leaf development and the relationship between LAI and NDVI). Simulated crop growth, particularly, LAI and NDVI were validated by measured LAI by LAI 2000 (LI–COR, USA) and measured NDVI by crop scan (S3 Fig). Overestimation of LAI in rainfed rice for the mid-season is due to drought-stress related effects, such as leaf rolling (visually observed), which UAV aerial photo derived LAI cannot capture as it was measured directly above the crop canopy by handheld crop-scan. In contrast, LAI in irrigated paddy field, was slightly underestimated, which is common in most of UAV studies due to reflection of irrigated water [22].

### Modeling and partitioning crop evapotranspiration

Crop evapotranspiration was calculated based on FAO 56 dual crop coefficient model, which is a modified version of Penman Monteith (1965) ET model [23]. The model estimates crop ET based on the reference crop evapotranspiration (ET_*0*_) multiplied to the sum of the transpiration coefficient (K_*cb*_) and the evaporation coefficient (K_*e*_) [24, 25].

$$\mathrm{ET}=(K_{\mathrm{cb}}+K_{e})\times {\mathrm{ET}}_{0}$$ (3)

where ET is the crop evapotranspiration, K_*cb*_ is the transpiration coefficient equivalent to the ratio of transpiration to potential evapotranspiration, K_*e*_ is the evaporation coefficient equivalent to the ratio of soil evaporation to potential evapotranspiration, ET_*0*_ is the reference evapotranspiration of a well-watered and healthy grass layer.

#### Calculation of K_cb_

In the FAO 56 dual crop coefficient approach of Allen [23], the basal crop coefficient or transpiration coefficient (K_*cb*_) is calculated based on seasonal change in vegetation ground cover. Estimates of K_*cb*_ for several crops including rice is provided as a K_*cb*_ curve with four growth stages (initial, development, mid-season, and late season) and it is recommended to use the estimated K_*cb*_ values after specific climatic adjustment.

Instead of applying the theoretical dual crop coefficient K_*cb*_ values of original FAO 56 model, we developed a daily basal crop coefficient (K_*cb*_) curve representing the actual crop growth and development. Following Choudhury [26] we derived the daily K_*cb*_ based on the daily and high resolution NDVI of the whole field:

Kcb=1‑[NDVImax‑NDVINDVImax‑NDVImin]kk* (4)

where NDVI_max_, NDVI_min_ and NDVI are vegetation indices for dense canopy, bare soil and normal vegetation respectively, *k* is a damping coefficient derived from the correlation of LAI and the ratio of canopy transpiration to potential evapotranspiration, k* is a damping coefficient derived from correlation of LAI and NDVI. The relationships between the ratio of unstressed transpiration (T) to reference crop evapotranspiration (ET_*0*_) and leaf area index (LAI), relationships between LAI and vegetation indexes has been shown [26–28]. Damping coefficient *k* is the coefficient derived by exponential correlation of the ratio of calculated daily T to reference ET_*0*_ and LAI while damping coefficient k* is the coefficient derived by exponential correlation of LAI and NDVI (S1 and S2 Figs).

#### Calculation of K_*e*_

The evaporation coefficient (K_*e*_) was calculated according to Allen [23]. K_*e*_ is maximal when the topsoil is wet or flooded and K_*e*_ is minimal to zero when the topsoil is dry. The upper limit of K_*c*_ (K_*cmax*_) which is an upper limit of evaporation and transpiration from cropped surfaces need to be defined before calculating K_*e*_ since the evaporation rate never fully amounts to total evapotranspiration and K_*e*_ needs to be limited by K_*cmax*_.

$$K_{\mathrm{cmax}}=\max\left(\{1.2+[0.04(u_{2}‑2)‑0.004({\mathrm{RH}}_{\min}‑45)]{\left[\frac{h}{3}\right]}^{0.3}\},(K_{\mathrm{cb}}+0.05)\right)$$ (5)

where K_*cmax*_ is the upper limit of evaporation and transpiration from a cropped surface, *u*_*2*_ is wind speed (ms^-1^), RH_*min*_ is the minimum relative humidity and K_*cb*_ is the transpiration coefficient derived by Eq (4).

The soil evaporation process is assumed to be controlled by 2 stages: Stage 1: an energy limiting stage and Stage 2: a falling-rate stage [23, 29]. Soil evaporation reduction coefficient (K_*r*_) is 1 when the soil surface is wet; K_r_ decreases when water content in the topsoil is limiting, and K_*r*_ becomes zero when total evaporable water (TEW = maximum amount of water that can be evaporated) in the topsoil is depleted. TEW for a complete drying cycle was estimated as:

$$\mathrm{TEW}=1000(\theta FC‑0.5\;\theta \;WP)*Z_{e}$$ (6)

where *TEW* is maximum depth of water that can evaporated from the soil when topsoil is completely wetted (mm), *θFC* is soil water content at field capacity (m^3^m^-3^), *θWP* is soil water content at wilting point (m^3^m^-3^) and Z_*e*_ is depth of surface soil layer (0.1 m). K_*r*_ for paddy rice is fixed at 1 since soil surface is flooded most of the time and soil surface is wet even during the drainage period. K_*r*_ of rainfed rice was calculated as:

$$K_{r}=(\mathrm{TEW-}D_{e,i‑1})/(\mathrm{TEW-REW})$$ (7)

where K_*r*_ is the soil evaporation reduction coefficient dependent on soil water depletion, D_*e*, *i-1*_ is the cumulative depth of evaporation depletion from topsoil at the end of the day (i-1), *TEW* is the total evaporable water (mm) calculated by Eq (6) and *REW* is the readily evaporable water which is cumulative depth of depletion of evaporable water from the soil surface layer at the end of stage one. During stage one drying, K_*r*_ is 1 and during stage two drying, Kr is 1 when D_*e*, *i-1*_ ≤ REW).

Finally, the evaporation coefficient (K_*e*_) is calculated as:

$$K_{e}=K_{r}(K_{\mathrm{cmax}}‑K_{\mathrm{cb}})\leq \mathrm{FEW}*K_{\mathrm{cmax}}$$ (8)

where K_*e*_ is the soil evaporation coefficient, K_*r*_ is the evaporation reduction coefficient, K_*cmax*_ is the maximum value of K_*c*_, FEW is the fraction of soil surface exposed and wetted. FEW is estimated based on the approximate fraction of exposed soil surface (*1-f*_*c*_) and limited with the fraction of the soil surface wetted by precipitation (for rainfed dryland) and irrigation (for flooded paddy (*f*_*w*_). Thus:

$$\mathrm{FEW}=\min(1‑f_{c},f_{w})$$ (9)

#### Calculation of *ET*_*0*_

ET_*0*_ is calculated by the Penman Monteith equation modified by Allen [23, 30].

$$\lambda {\mathrm{ET}}_{0}=\frac{\Delta (R_{n}‑G)+((\rho C_{p}(e_{s}‑e_{a}))/r_{a}}{\Delta +\gamma (1+(\frac{r_{c}}{r_{a}}))}$$ (10)

where λ is the latent heat of vaporization of water vapor,Δ is the slope of the saturation vapor pressure temperature relationship, R_*n*_ is the net radiation, G is the soil heat flux which is ignored for daily calculation as suggested by Allen [23], e_*s*_-e_*a*_ is the vapor pressure deficit of the air, ρ is the mean air density at constant pressure, C_*p*_ is the specific heat of the air, r_*a*_ is aerodynamic resistance, r_*c*_ is the canopy resistance and γ is the psychrometric constant. All the parameters except the canopy resistance (r_*c*_) were set at default values recommended by Allen [23].

In our case, instead of hypothetical parameters for grass canopy provided by FAO 56 dual crop coefficient model, we used the measured crop physiological parameters (leaf resistance to water vapor transfer, and plant height, which was used to estimate aerodynamic resistance) for well-irrigated and healthy rice in the field. Therefore, our ET_*0*_ was reference crop evapotranspiration of rice under standard crop management.

We calculated canopy transpiration (T) by Eq (9) but used the net radiation intercepted by the crop canopy (R_*nsC*_) instead of net solar radiation (R_*n*_). To estimate R_*nsC*_, we partitioned incoming net radiation (R_*n*_) to R_*nsC*_ (net radiation intercepted by crop canopy) and R_*nss*_ (residual net radiation reaching the soil surface). R_*nss*_ was calculated according to Beer’s law [31]:

$$R_{\mathrm{nss}}=R_{n}*exp(‑C_{r}LAI)$$ (11)

where C_*r*_ is the extinction coefficient of the vegetation for net radiation and is in the range of 0.5 to 0.7; 0.6 was applied in our case [32, 33]. Simulated daily ET of rainfed rice was verified by chamber measured ET. The paddy rice ET model was validated by applying measured ET and NDVI of monsoon 2012 paddy rice, at Haean, South Korea (Lee, unpublished).

### Modeling and partitioning daily carbon fluxes

Gross primary production on a daily basis throughout the growing season was modelled based on canopy light use efficiency (LUE), daily NDVI and PAR:

GPP=LUE×NDVI×PAR (12)

where GPP is the gross primary production, LUE is the canopy light use efficiency, NDVI is the normalized vegetation index and PAR is the photosynthetic active radiation [34, 35]. Light use efficiency (LUE) was obtained from hyperbolic light response curves (rectangular hyperbola model) of chamber based GPP estimates [36]. Chamber based GPP was derived from measured NEE and R_eco_ (see section “Canopy carbon and water flux measurement”).

Daily ecosystem respiration of rainfed and paddy rice was calculated following Reichstein [37] as:

$$R_{\mathrm{eco}}=R_{\mathrm{ecoref}}\times f(T_{\mathrm{soil}})\times g(\mathrm{SWC})$$ (13)

where *g*(SWC) is the saturation function [38, 39]; R_*ecoref*_ is reference ecosystem respiration, *f*(T_*soil*_) is the function developed by Lloyd and Taylor [40] as:

$$f(T_{\mathrm{soil}})=e^{E_{0}(\frac{1}{T_{\mathrm{ref}}‑T_{0}}‑\frac{1}{T_{\mathrm{soil}}‑T_{0}})}$$ (14)

where T_*ref*_ and T_*0*_ are fixed to 15 and -46°C, respectively, T_*soil*_ is the soil temperature at 5cm depth, E_*0*_ is the activation energy and was considered to be a free parameter. Simulated CO_2_ fluxes (NEE, GPP and R_eco_) were compared to chamber based estimates of NEE, GPP and R_eco_ at different crop growth stages. Chamber observations of three different plots for a specific measurement day were pooled so that the measured observations could better represent the whole field.

The productivity of paddy and rainfed rice system was assessed by calculating agronomic and ecosystem water use efficiency (WUE). Agronomic WUE (WUE*agro*) is defined as the ratio of biomass production (grain yield) per amount of evapotranspiration (ET):

$${\mathrm{WUE}}_{\mathrm{agro}}=\frac{\mathrm{grain}\;\mathrm{yield}}{\mathrm{ET}}$$ (15)

Grain yield of paddy and rainfed rice was estimated based on 1000-grain-weight of oven dried (moisture percent of dried grain = ~14%) harvested samples (n = 6). 1000-grain- weight is regarded as a standard and stable parameter for the yield estimation of crop and is the total grain weight of the oven-dried 1000 grains.

Ecosystem water use efficiency (WUE*eco)* is defined as the ratio of gross primary production (GPP) to evapotranspiration (ET):

$${\mathrm{WUE}}_{\mathrm{eco}}=\frac{\mathrm{GPP}}{\mathrm{ET}}$$ (16)

Including both respiratory carbon fluxes (R_*eco*_) and ecosystem productivity (GPP), ecosystem WUE can also be defined as the ratio of net ecosystem carbon exchange (NEE) to ET:

$${\mathrm{WUE}}_{\mathrm{NEE}}=\frac{\mathrm{NEE}}{\mathrm{ET}}$$ (17)

To exclude day to day effects of changing vapor pressure deficit and highlight the impact of seasonal changes in water availability on WUE, VPD is often included in the equation [17, 41], however we did not find any significant VPD effects on the calculations of WUE during our monsoon 2013 field study in S. Korea.

To quantify the impact of unproductive water loss (E) and respiratory carbon loss (R_*eco*_), we also calculated productive WUE*agro* (the ratio of yield to transpiration (T)) and productive WUE*eco* by excluding evaporative losses (the ratio of GPP to T). We also calculated WUE*eco* as the ratio of NEE/T to highlight the influence of evaporation on WUE*eco* of paddy rice.

### Statistical analysis

Two statistical tests were used to evaluate the model performance of daily NDVI, LAI, ET, GPP and R_*eco*_ simulation: i) root mean square error (RMSE) and ii) model efficiency (ME) [42].To test for a relationships between daily average environmental variables (Radiation, T_*air*_, T_*soil*_, VPD, SWC) and measured canopy fluxes (sum of day time NEE, GPP, R_*eco*_, ET), a Spearman rank order correlation was performed. One way ANOVA followed by a post-hoc test (TukeyHSD) was applied to assess the differences in ecosystem carbon exchange (NEE, GPP, R_*eco*_), ET and grain yield, WUE*eco* and WUE*agro* between rainfed and paddy rice. Homogeneity of variance was tested by Bartlett test and no data transformation had to be applied to meet the requirements of ANOVA. All statistical analysis were performed using R statistical software version 3.1.2 [43].

The uncertainty of FAO 56 dual crop approach was previously reported although it considers the most environmental variables and crop factors, which many other models do not [44, 45]. We tested the performance of our FAO 56 dual crop and *NDVI K*_*cb*_ method against other well know ET estimation methods (see S1 File). For the simulated daily transpiration, we cross checked with measured leaf transpiration of both paddy and rainfed rice, which showed a similar tendency (see S1 File).

## Results and discussion

### Climate and rice growth

**Fig 1:**
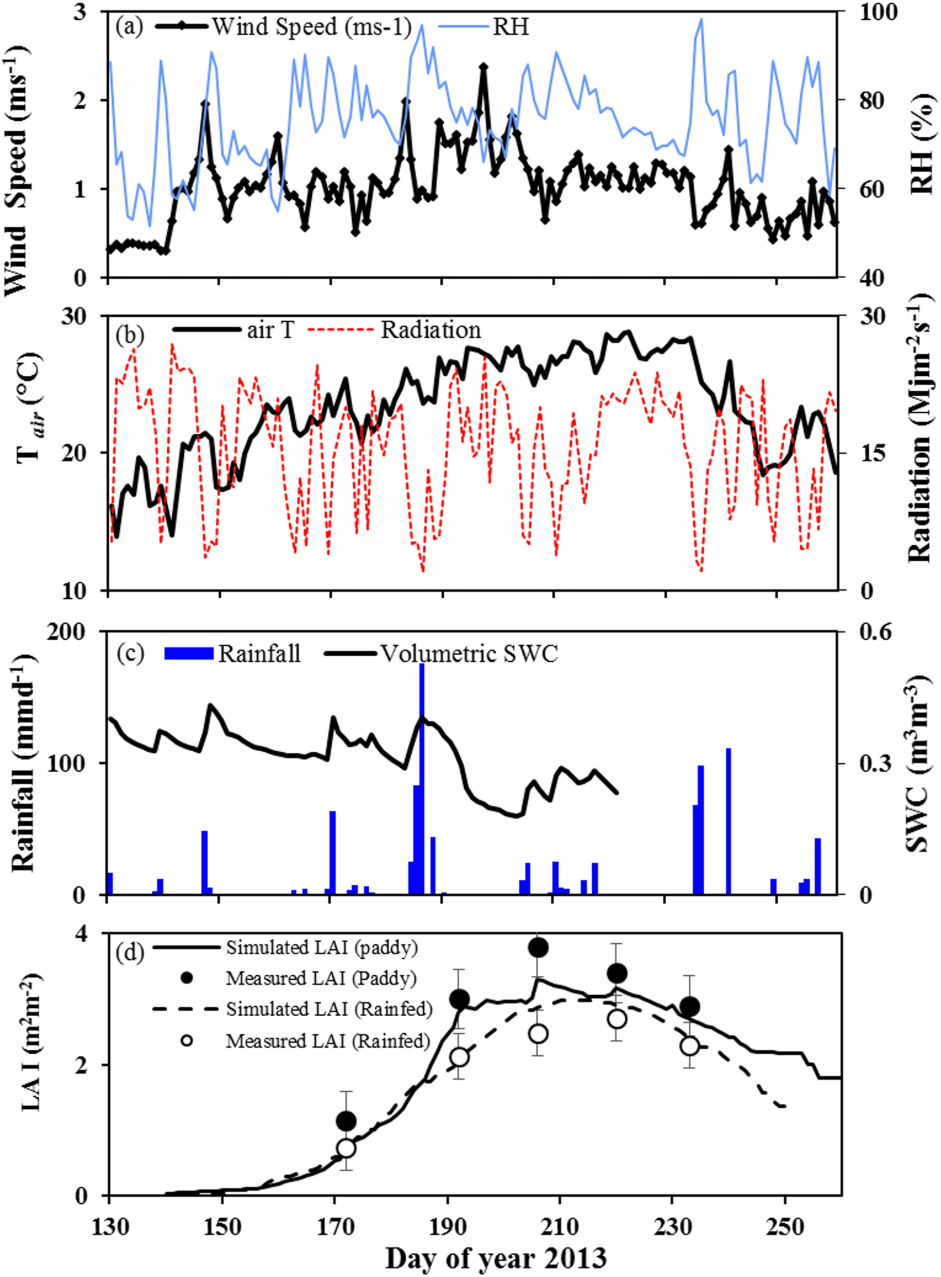
Climate conditions and rice growth during the monsoon 2013. (a) Daily averages of windspeed (ms^-1^) and relative humidity (%); (b) daily averages of air temperature (°C) and radiation (Mjm^-2^s^-1^); (c) daily total rainfall (mmd^-1^) and daily average volumetric soil water content at 5cm depth (m^3^m^-3^); (d) daily LAI of paddy (solid line) and rainfed (dashed line); lines represent simulated LAI and circles represent measured LAI (Black circle = measured LAI of paddy, white circle measured LAI of rainfed rice, n = 9, mean values ± SD).

The weather conditions of the study area generally followed the typical East Asian temperate monsoon climate system. Annual total rainfall of 1332 mm in 2013 was slightly less compared to 30 years annual average of 1391 mm (1981–2010). There was a dry period with almost no rainfall between DOY 190 and 202 which resulted in very low (0.18 m^3^m^-3^) volumetric soil water content. However, due to the high intensity of single rain events, the total precipitation amounted to 973 mm during the rice growing season (i.e., DOY 130 to 255 or May to September) which was above the long-term average of 799 mm for the same time period. Daily solar radiation was up to 26.9 MJm^-2^s^-1^ in May (DOY 141) but declined from the end of June (DOY 175) to a low of 2.0 MJm^-2^s^-1^ in July (DOY 194) with 5 ± 1.8 sunshine hours per day. Daily mean, air temperature (T_*air*_) during the rice growing season were 23.4°C. T_*air*_ measured over the crop canopy was 5.27 ± 2.20°C lower in paddy rice than in rainfed rice. The highest midday mean relative humidity (RH) was 98.31% occurring in August (DOY 236) and the lowest midday mean RH, 51.73% in May (DOY 137) (Fig 1A, 1B and 1C).

Both rainfed and paddy rice had similar trends of LAI although rainfed rice reached slightly lower LAI from the end of June onwards (Fig 1D). The peak growth for both rainfed and paddy rice was reached in August with a maximum plant height of 0.80 ± 0.97 m and 0.89 ± 0.66 (not shown), and LAI of 2.97 ± 1.21 m^2^m^-2^and 3.29 ± 0.65 m^2^m^-2^, respectively. Paddy rice yielded 6612 ± 218 kg grains ha^-1^while the grain yield of rainfed rice was 9.4% lower (5989 ± 683 kg grains ha^-1^) but differences between paddy and rainfed were statistically not significant (F = 1.515, p = 0.286).

### Carbon and water fluxes of paddy and rainfed rice

**Fig 2:**
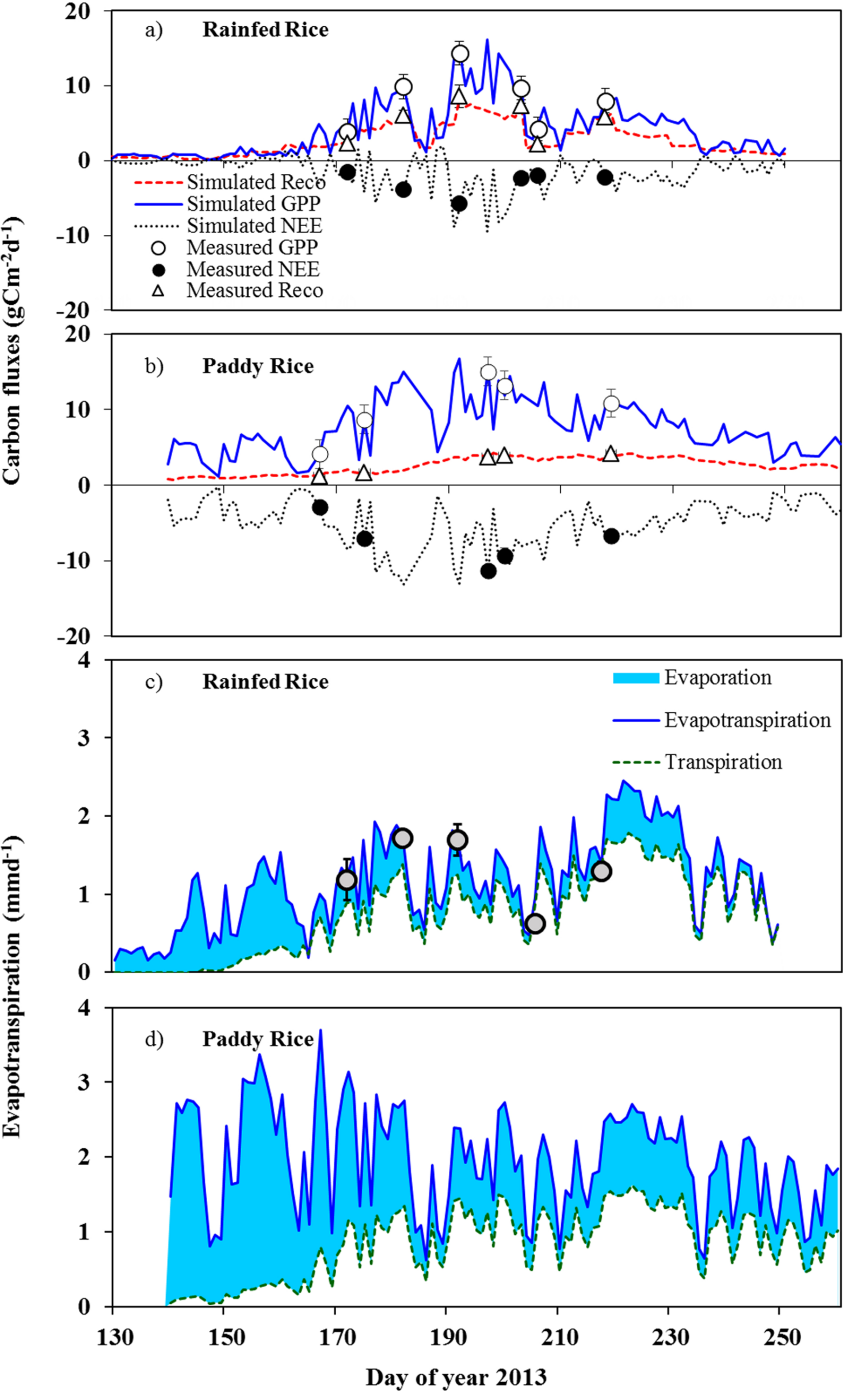
**Seasonal carbon and water fluxes of rainfed and paddy rice:** daily carbon fluxes of (a) rainfed rice; (b) paddy rice (simulated gross primary production, black line; measured gross primary production, black circle; simulated ecosystem respiration, red dashed line; measured ecosystem respiration, red triangle; simulated net ecosystem exchange, black dotted line; chamber measured net ecosystem exchange, white circle); daily water fluxes of (c) rainfed rice and (d) paddy rice(simulated daily evapotranspiration, blue line; chamber measured evapotranspiration, gray circle, simulated transpiration, green line; evapotranspiration minus transpiration (evaporation), blue shaded area) (n = 3, mean value ± SD for measured fluxes).

To investigate the role of carbon and water exchange on WUE*eco* we measured canopy gas exchange (NEE, GPP, R_*eco*_ and ET) at different growth stages. For the seasonal trend, we simulated daily NEE, GPP, R_*eco*_, ET, T and E. Our simulated values were validated against the chamber measured fluxes, showing a good agreement between measured and modeled data (NEE: ME = 0.86, RMSE = 0.58, R^2^ = 0.86; GPP: ME = 0.95, RMSE = 0.63, R^2^ = 0.99; R_*eco*_: ME = 0.72, RMSE = 0.51, R^2^ = 0.75; ET: ME = 0.82, RMSE = 0.13, R^2^ = 0.97) (Fig 2).

As expected from literature [14, 15], rainfed and paddy rice showed significantly different water and carbon fluxes (n = 3, F = 24.5, p ≤ 0.01; see S1 Table). Growing season total evapotranspiration (ET) of paddy rice was 40% higher than that of rainfed rice (F = 29.7, p ≤ 0.01). However, there was no significant difference between growing season total canopy transpiration (T) although T of paddy rice was 10% higher than that of rainfed rice (F = 0.23, p = 0.55). These differences were mainly caused by microclimatic differences, in particular in VPD (see Fig 1).

Although we studied both rice systems adjacent to each other under the same environmental conditions, canopy microclimate differences between paddy and rainfed rice were observed. Canopy air temperature (T_*air*_) of paddy was always lower than that of rainfed rice (by 5.27 to 2.20°C). Soil temperature (T_*soil*_) of paddy rice was lower than that of rainfed rice except during the maturity stage (DOY 230 onward) when the flooded water was drained from paddy field. Evapotranspiration of rainfed rice was mainly driven by T_*air*_, T_*soil*_ and VPD (Spearman’s ρ = 0.65, 0.57, 0.47, respectively, p ≤ 0.01) while that of paddy rice was driven by radiation and VPD (Spearman’s ρ = 0.87, 0.67, respectively, p ≤ 0.01).

**Fig 3:**
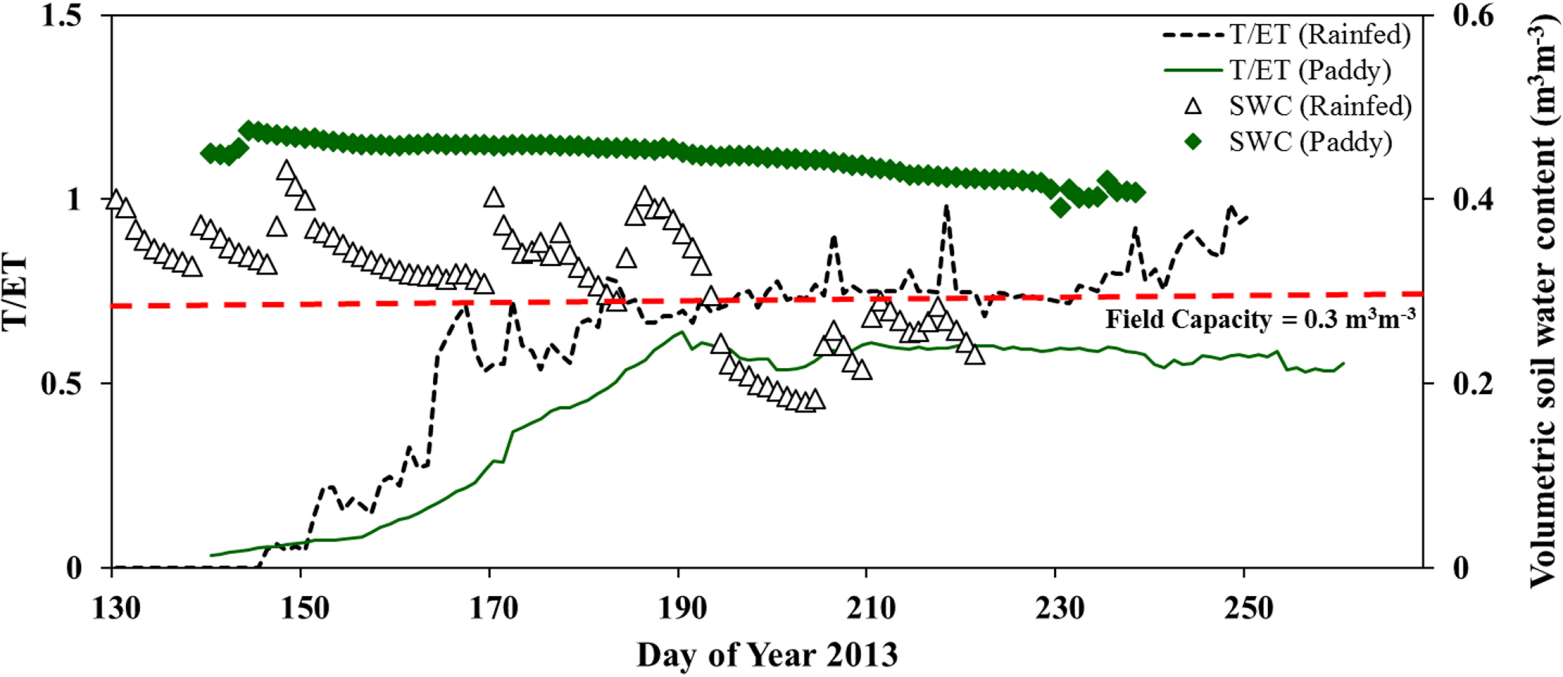
Seasonal change in the ratio of transpiration to evapotranspiration (T/ET) and in soil water content: Change in T/ET of rainfed rice (black dashed line) and paddy rice (green straight line) along with change in soil water content at 5 cm (white triangle = rainfed rice, green diamond = paddy rice). The ratio of transpiration to evapotranspiration (T/ET) was calculated based on simulated daily T and ET of monsoon 2013. T/ET less than zero and greater than one are not shown in the figure.

Daily contribution of transpiration to evapotranspiration (T/ET) of paddy and rainfed rice was calculated based on simulated daily T and ET. T/ET of paddy rice steadily increased with the increasing canopy density (LAI) (Fig 3).

T/ET of rainfed rice fluctuated with changes in soil water content (SWC) and the highest T/ET was found at SWC of 0.34 m^3^m^-3^ during seedling stage (on DOY 162) (Fig 3). The response of T/ET to decreasing SWC was strong until SWC declined below field capacity (0.30 m^3^m^-3^). When SWC decreased below the field capacity in rainfed and 0.4 m^3^m^-3^ in paddy rice, SWC was no longer the main determining factor driving T/ET. Instead, VPD and radiation were the factors driving T/ET of paddy rice (Spearman’s *ρ* = 0.72, respectively, p ≤ 0.01). H_2_O fluxes from rainfed rice was mainly dominated by transpiration (T/ET = 65%) while that of paddy rice was mainly driven by evaporation (T/ET = 40%). When soil water content (SWC) declined below field capacity, T contributed 80 to 90% of H_2_O flux in rainfed rice. Evaporative water loss (E) was dominant in the early vegetative stages (until DOY 200) in both paddy and rainfed rice. At the end of active tillering stage, along with the increasing canopy density, canopy T became the dominant water flux in both paddy and rainfed rice. Nevertheless, E of paddy rice was significantly higher than that of rainfed rice (F = 24.6, p ≤ 0.01).

**Fig 4:**
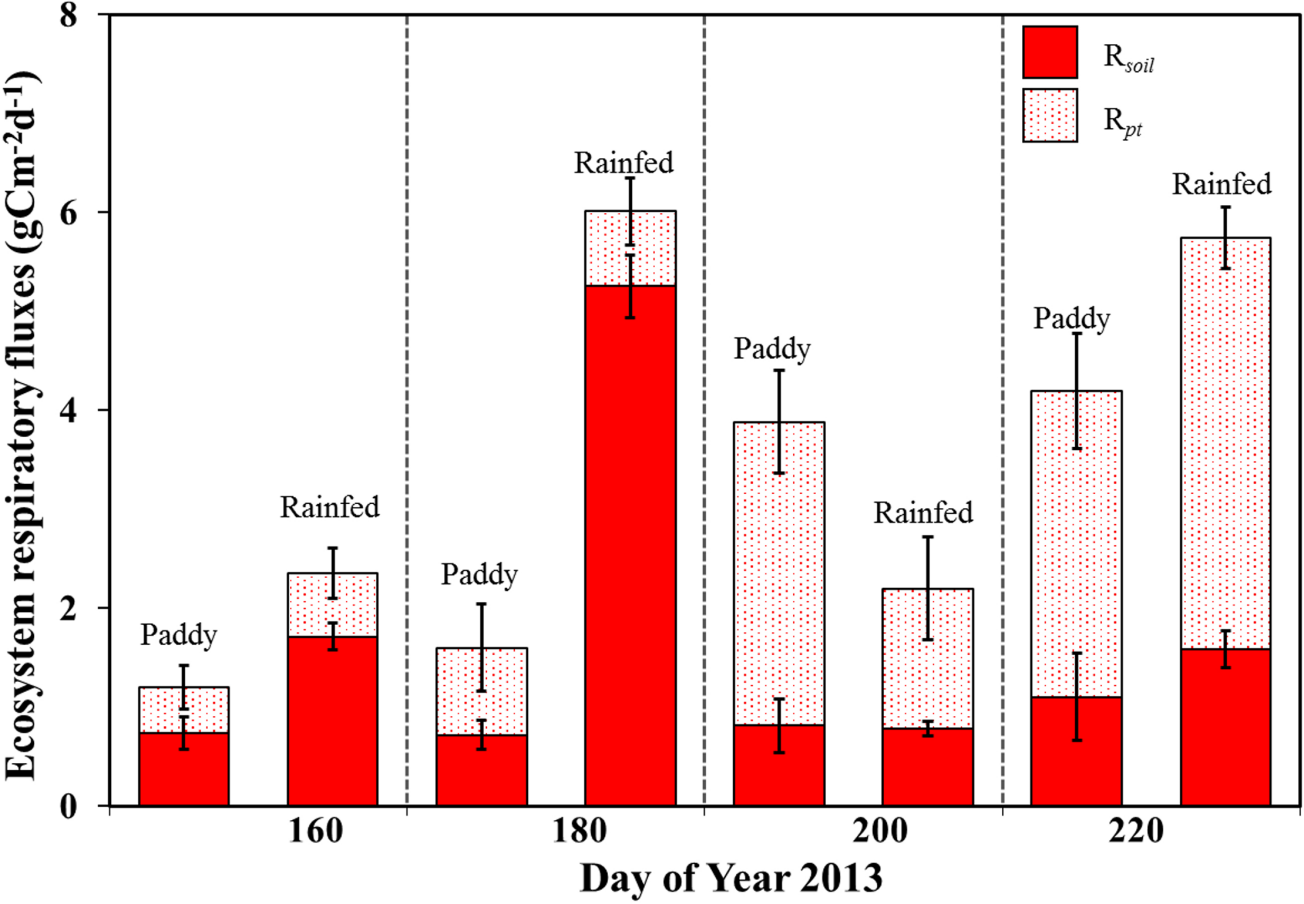
Contribution of soil and crop respiration of paddy and rainfed rice. Ecosystem respiratory flux partitioning of paddy and rainfed rice was done based on dark chamber measured soil respiration (R_*soil*_, Solid red) and dark chamber measured crop respiration (R_*pt*_). Measurements were carried out during seedling, tillering, heading and maturity stage (n = 3–4, mean value ± SD).

Growing season total gross primary production (GPP = sum of simulated daily GPP during monsoon rice growing season 2013) of paddy and rainfed rice were not significantly different. However, paddy rice had significantly lower ecosystem respiration (R_*eco*_) in both, chamber measured and simulated daily R_*eco*_, hence net ecosystem exchange (NEE) was higher in paddy rice (Figs 2 and 4). Lower R_*eco*_ in paddy rice could be due to its flooded anaerobic condition, which is not much favorable for soil microorganisms compared to aerobic condition of rainfed rice. Moreover, it could also be due to the loss of CO_2_ respired by roots and soils in the flooded water as explained in [46]. Throughout the growing season, R_*eco*_ and GPP variations were in synchrony in rainfed rice while they were not in paddy rice. This could be due to several biophysical factors but most likely the dominant factor for the dryland rainfed rice seasonal variations in both carbon fluxes were soil moisture seasonal variations [47].

Growing season total ecosystem respiratory carbon loss in rainfed rice was 48.78% of net carbon fluxes while paddy rice ecosystem respiratory carbon loss was only 33.65% of net fluxes. Both measured and simulated R_*eco*_ of rainfed and paddy rice was strongly correlated to T_*air*_ and T_*soil*_ (Spearman’s *ρ* = 0.74, 0.80, respectively for paddy; Spearman’s *ρ* = 0.74, 0.80, respectively for rainfed, p ≤ 0.01, n = 12). According to dark chamber measured soil and plant respiration, R_*eco*_ of paddy rice was dominated by plant respiration (R_*pt*_) while R_*eco*_ of rainfed rice was mainly dominated by soil respiration (R_*soil*_), particularly until doy 200 (Fig 4, see also S2, S3 and S4 Tables). Therefore, higher respiratory carbon loss of rainfed rice system was clearly due to its higher soil respiration.

### Trade-off between evaporative and respiratory losses in paddy and rainfed rice

The main goal of this study was to compare carbon and water fluxes between conventional paddy and rainfed rice farming analyzing the distinct contributions of unproductive water losses from soil evaporation and respiratory carbon losses to net ecosystem carbon and water exchange. Quantifying the impact of these distinct irrigation treatments is specifically important to find an optimal balance between low evaporative and respiratory losses for a sustainable rice production. We found different carbon exchange pattern of water saving rainfed rice compared to conventional paddy rice clearly. Since the paddy rice system is the conventional rice production system, which can be found in most of the global rice production area [2, 11], significantly reduced evaporation per unit production area can raise a question on possible global or regional water cycle changes if the majority of paddy systems were converted to water saving production systems [48–50]. Since global water and carbon fluxes are coupled by vegetation, impacts on water cycle could lead to impacts on the carbon balance [51, 52].

**Fig 5:**
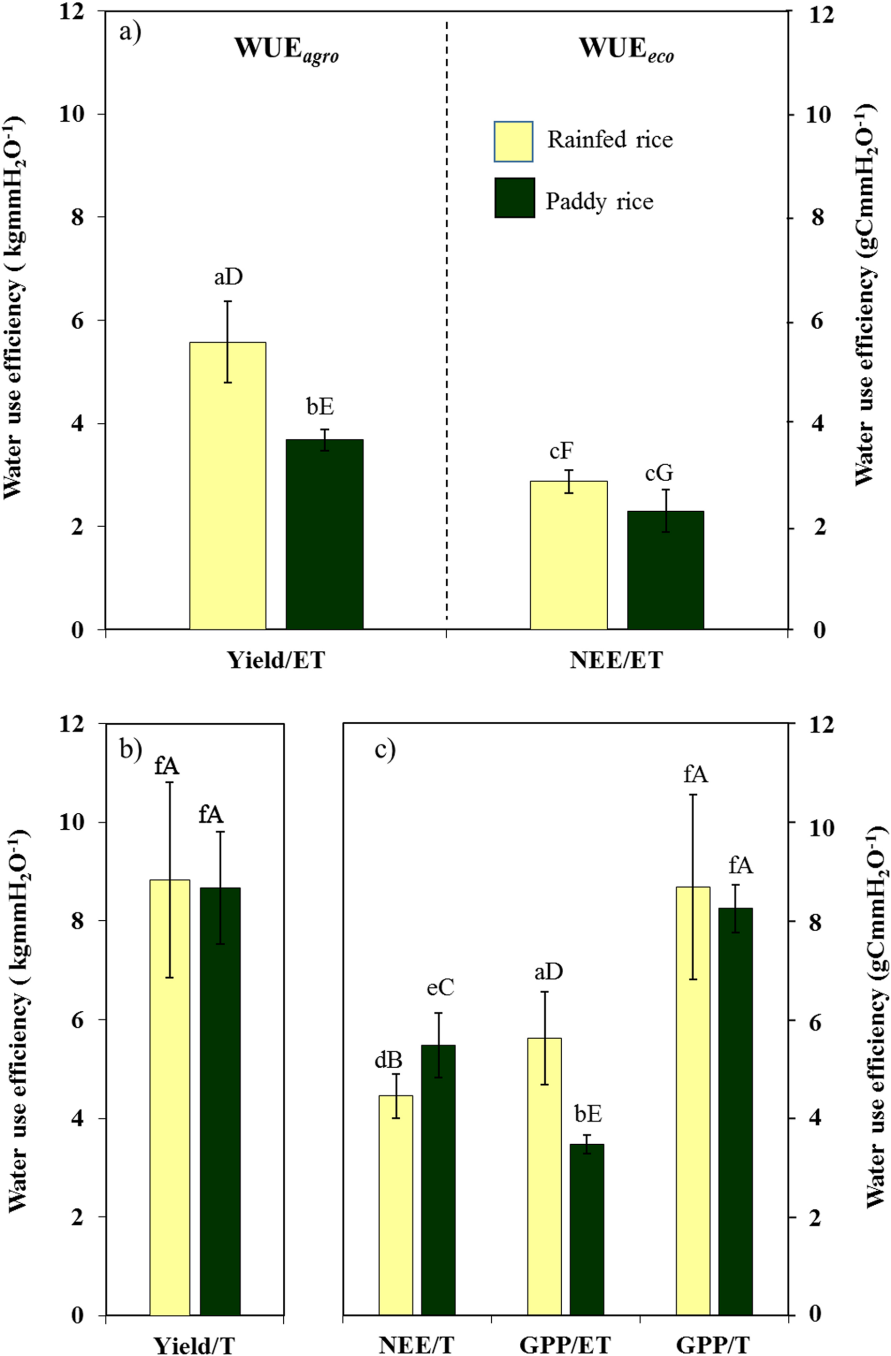
Effects of respiratory carbon loss and evaporative water loss over water use efficiency. (a) Yield/ET of rainfed rice (light yellow) was higher than paddy rice(dark green) (F = 10.61, p ≤ 0.05)but NEE/ET were not significantly different, (b) Yield/T was not significantly different between paddy and rainfed rice (F = 0.14, p = 0.75) highlighting the higher evaporative loss in paddy rice; (c) Lower GPP/ET of paddy rice due to its higher evaporative water loss (F = 9.96, p ≤ 0.05); and higher GPP/ET of rainfed rice due to its higher respiratory carbon loss (F = 25.41, p ≤ 0.01).(One way ANOVA followed by TukeyHSD *posthoc* test was applied to access WUE differences between paddy and rainfed (n = 3–12, mean value ± SD); different small letters denote significant differences among paddy and rainfed rice within each panel (a to f) while different capital letters denote significant differences among different water use efficiencies (A to G).

As expected, we clearly found higher WUE*eco* and WUE*agro* (GPP/ET and Yield/ET) in rainfed compared to paddy rice [15, 16, 22] (Fig 5). However, a different picture emerged when considering the productive water use and respiratory losses. Generally, WUE*eco* is defined as the ratio of gross primary production to evapotranspiration (WUE*eco* = GPP/ET) and has been estimated for different ecosystems ranging from grasslands to cultivated vegetation often without considering the influence of respiratory carbon losses (R_*eco*_)[37, 41]. Although, this yields information on the water use efficiency of plants to fix carbon at the stand level, considering ecosystem respiration into WUE assessments is crucial to gain an ecosystem perspective [17]. Accordingly, higher GPP/ET in rainfed rice ecosystem was due to higher R_*eco*_ since rainfed rice had similar GPP to paddy rice but lower net ecosystem carbon exchange (NEE). As GPP was derived based on measured Light Use Efficiency (LUE), which is almost constant for the same crop species, the similarity of GPP of paddy and rainfed rice could be explained by the similar LUE trends of both rainfed and paddy rice system, which was the same rice variety. LUE of both rainfed and paddy system reached their maximum during the late vegetative growth stage (i.e., DOY 170 to 190) (unpublished data) and declined thereafter, following the similar seasonal and phenological tendency reported by Inoue [12]. Therefore, the higher WUE*eco* (GPP/ET) of rainfed rice system was mainly linked with its higher R*eco* and accounting for this difference by considering net ecosystem exchange (NEE/ET) yielded comparable water use efficiencies of both rice production systems.

**Fig 6:**
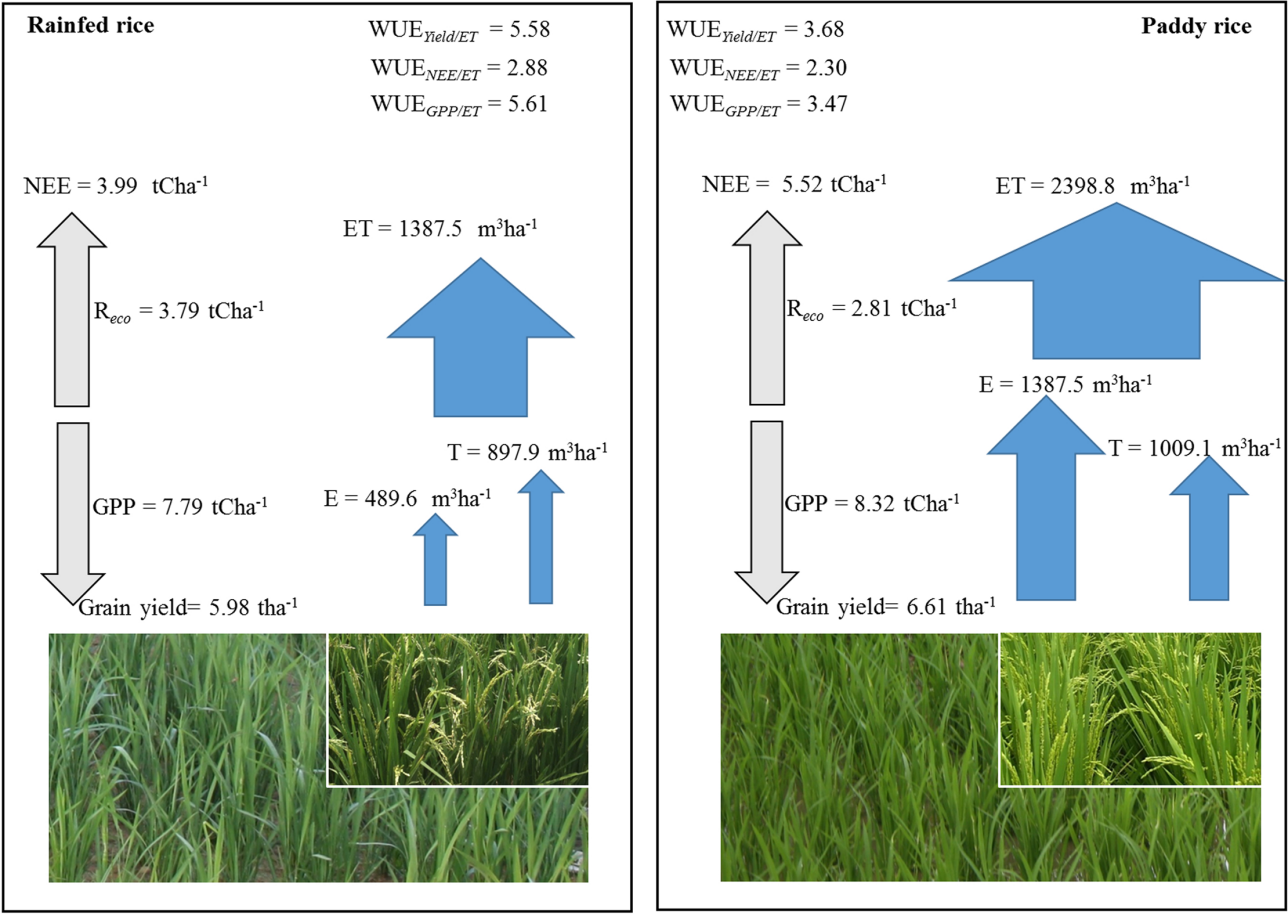
Seasonal carbon and water balance of paddy and rainfed rice. Seasonal cumulative gross primary production (GPP), net ecosystem exchange (NEE), ecosystem respiration (R_*eco*_), evapotranspiration (ET), transpiration (T) and grain yields were used in this schematic representation. All data except the grain yield (grain yield: n = 6 ± SD) were calculated based on the daily simulation presented in Fig 2. Crop growing season was 120 days (sowing to harvest).

Along with respiratory carbon loss, unproductive water loss, i.e. soil evaporation (E) considerably affects the water use of rice production. Evaporation (E) influences T by influencing canopy microclimate (canopy temperature and VPD) which indirectly influences T/ET, water use, crop growth and yield [15, 53, 54]. As a result of the maximization of carbon gain per water use along with available water, productive water use efficiency (GPP/T and Yield/T) of paddy and rainfed rice were almost equal. However, T/ET was significantly different between paddy and rainfed rice (Fig 3) and increased along with crop growth. By contrast, the contribution of evaporation to H_2_O fluxes was relatively similar in both production types from the end of tillering stage onwards (DOY 200) when the crop canopy was dense, as major differences in unproductive water loss occurred before canopy closure. Overall, the higher WUE*agro* of rainfed was in concert with significantly reduced evaporation but also a slightly decreased grain yield compared to paddy (Fig 6).

According to water scarcity projections based on different parameters (for example, socioeconomic assumptions, climate change scenarios, etc.), the frequency of extreme droughts may increase in many regions (Eastern China, India, Western Europe and Middle East) [47, 55], highlighting the change in regional specific hydrologic cycle. Moreover, global annual yield increase rates (current rate = 2.4%) of major agricultural crops (especially, rice, maize, wheat, soybean) should be doubled to meet the projected food demand by 2050 [56, 57]. Hence, a choice between the trade-off of paddy rice production with high evaporative losses and methane emissions and rainfed rice with increased respiratory losses and possible impact on grain yield [58, 59], depends largely on the regional water availability and precipitation regime.

### Taking into account other greenhouse gases

The overall greenhouse gas balance of conventional flooded rice system is still not entirely resolved. Since CH_4_ and N_2_O emission not only depend on the amount of flooding but also on other factors such as climatic conditions, crop growth, atmospheric CO_2_ concentration [60, 61], source and rate of fertilizer applied [62], inorganic and organic carbon substrate availability for denitrifying bacteria, oxygen availability and bacterial activity [63]. Traditional flooded paddy rice has high CH_4_ and low N_2_O emissions while non-flooded rainfed rice shows low CH_4_ but high N_2_O emissions [9] together with high respiratory CO_2_ release, all being relevant greenhouse gases [64]. CH_4_ is a 25 times more potent greenhouse gas than CO_2_ according to a 100-years timescale global warming potential of IPCC [65]. If a policy that favors the conversion of conventional paddy rice production to rainfed rice production will be proposed for a certain region, from the perspective of reducing CH_4_ emission, we need to counterbalance the CH_4_, CO_2_ and N_2_O emissions of both rice production systems. For example, in our case, rainfed rice released 980 Kg C ha^-1^season^-1^ more CO_2_ than paddy rice system during one growing season (Fig 6, Ecosystem respiration (*R*_*eco*_); 1 ton = 1000 Kg). Given the fact that CH_4_ is 25 times higher potent than CO_2_ in terms of global warming potential, an increase in 980 KgCha^-1^season^-1^ CO_2_ due to the conversion of paddy rice to rainfed rice system must be counterbalanced with a minimum 40 Kg CH_4_ ha^-1^ decrease in CH_4_ emission. Wassmann [61] compared the CH_4_ emission of paddy and rainfed rice systems and pointed out that irrigated paddy rice emit 40 to 80 KgCH_4_season^-1^ more than rainfed rice. Thus, if the environment, socioeconomic and other regional/local needs (e.g., water demand and water scarcity, etc.) favor the conversion of conventional paddy rice to rainfed rice farming, this policy shift is doable by a careful counterbalance of changes in CH_4_, CO_2_ and N_2_O efflux of two different systems. Under the environmental conditions at the present study location, with abundant monsoon rainfall, the high water consumption of paddy rice presents much less of concern than high respiratory losses of rainfed rice. However, in different climates, such as the Mediterranean, Africa or Middle East, producing rice in a more sustainable water management regime considering its impact on the regional hydrological cycle may well outweigh slight impacts on grain yield and higher respiratory losses.

## Conclusion

We cultivated the same rice variety under the same environmental conditions except water available and observed significant differences in ecosystem carbon and water balance. Significantly higher WUE*agro* (yield/ET, 34% higher) was found in rainfed compared to paddy rice, as expected. However, these differences were mainly caused by a 65% higher water loss via soil evaporation, especially before canopy closure. From an ecological perspective, the higher evaporative water loss under paddy conditions was fully compensated by higher respiratory carbon losses under rainfed conditions, leading to similar *WUE*_*eco*_ (NEE/ET). Also, canopy WUE (GPP/T) was similar under both water management regimes, indicating that rice plants did not significantly shift their physiological control on transpiration and photosynthesis.

Therefore, implications for future changes in management practices depend on regional water availability and the trade-off between changes in ecosystem CO_2_ release vs. other greenhouse gases (CH_4_, N_2_O). Our results suggest that a transition from rainfed to paddy water management following canopy closure is a good comprise from agronomic and ecological perspectives, as excessive evaporative water losses under rainfed management occur primarily in the early growth stages.

## Supporting information

S1 Fig LAI and T/ET_*o*_ relationship. Relationship between T/ET_*o*_ and LAI of (a) paddy and (b) rainfed rice.LAI was calculated as leaf area per ground area where Leaf area (LA) was determined with a Leaf Area Meter (LI−3000A, LI−COR, USA). T/ET_*o*_ was calculated as the ratio of estimated daily transpiration of the LAI measurement date (Eqs 3 and 6) to estimated reference evapotranspiration (Eq 3).
(PNG)

S2 Fig LAI and NDVI relationship. Relationship between NDVI and LAI of (a) paddy rice and (b) rainfed rice. LAI was calculated as leaf area per ground area where Leaf area (LA) was determined with a Leaf Area Meter (LI−3000A, LI−COR, USA). NDVI was measured by Cropscan (Cropscan Inc., USA).
(PNG)

S3 Fig Simulated daily crop growth. Simulated daily crop growth of paddy and rainfed rice a) LAI, b) NDVI, c) Dual crop coefficient (K_*cb*_). Daily K_*cb*_ was simulated based on daily NDVI, after following Choudhury 1994.
(PNG)

S4 Fig Simulated canopy transpiration. Simulated canopy transpiration followed the seasonal trends of measured leaf transpiration (Mean ± SE) of (a) rainfed rice (b) paddy rice.
(PNG)

S5 Fig GRAMI rice crop model flow diagram adapted from (Maas, 1995; Ko et al., 2015.). (PNG)

S1 Table Field management activities and timeline in DOY. (DOCX)

S2 Table Water and carbon fluxes, grain yield and water use efficiency of paddy and rainfed rice. Net Ecosystem Exchange (NEE, -NEE = GPP+ R_*eco*_) is the balance between photosynthetic uptake and release of carbon dioxide by autotrophic and heterotrophic respiration. Gross primary production (GPP) is photosynthetic uptake. Ecosystem respiration (R_*eco*_) is respiration from soil and plant. Ecosystem water use efficiency was calculated as the ratio of NEE to evapotranspiration (ET); the ratio of NEE to transpiration (T) and the ratio of GPP to T. Agronomic water use efficiency was calculated as the ratio of grain yield to ET. Differences between paddy and rainfed were tested by one way ANOVA: Carbon, water fluxes, Grain yield and water use efficiency were compared not only as crop seasonal sum but also as growth stage specific.
(DOCX)

S3 Table Correlation matrix of carbon and water fluxes and environmental variables of paddy rice. (DOCX)

S4 Table Correlation matrix of carbon and water fluxes and environmental variables of rainfed rice. (DOCX)

S5 Table Comparison of different crop *ET* estimation methods. *Mk, PT, 56PM, m56PM_80_, m56PM_100_, m56PM_120_* are conventional reference crop *ET* (*ET_0_*, grass as reference crop) estimation methods while *m56PM_mrc_* is reference crop *ET* of rice (*ET_0_*, healthy and well-watered rice as reference crop). *K_cb_FAO_* is the FAO recommended hypothetical basal crop coefficients (Provided in section (4.2.1), Table (4.2) while *K_cb_NDVI_* is *NDV*I derived basal crop coefficient. R^2^ is determination of coefficients, *RMSE* is root mean square error, p (t-test) is level of significant of the test, CV (*RMSE*) is coefficient of variation determined by *RMSE, ME* (Nseff) is model efficiency and Score is the score of model performance ranked based on *ME* and R^2^.
(DOCX)

S1 File Calculations of net radiation and ET. (DOCX)
