# Supplementary figures and images for: Quantifying differences in water and carbon cycling between paddy and rainfed rice (Oryza sativa L.) by flux partitioning
Source: PLoS One. 2018 Apr 6;13(4):e0195238. doi: 10.1371/journal.pone.0195238 (PMC5889072; doi:10.1371/journal.pone.0195238)

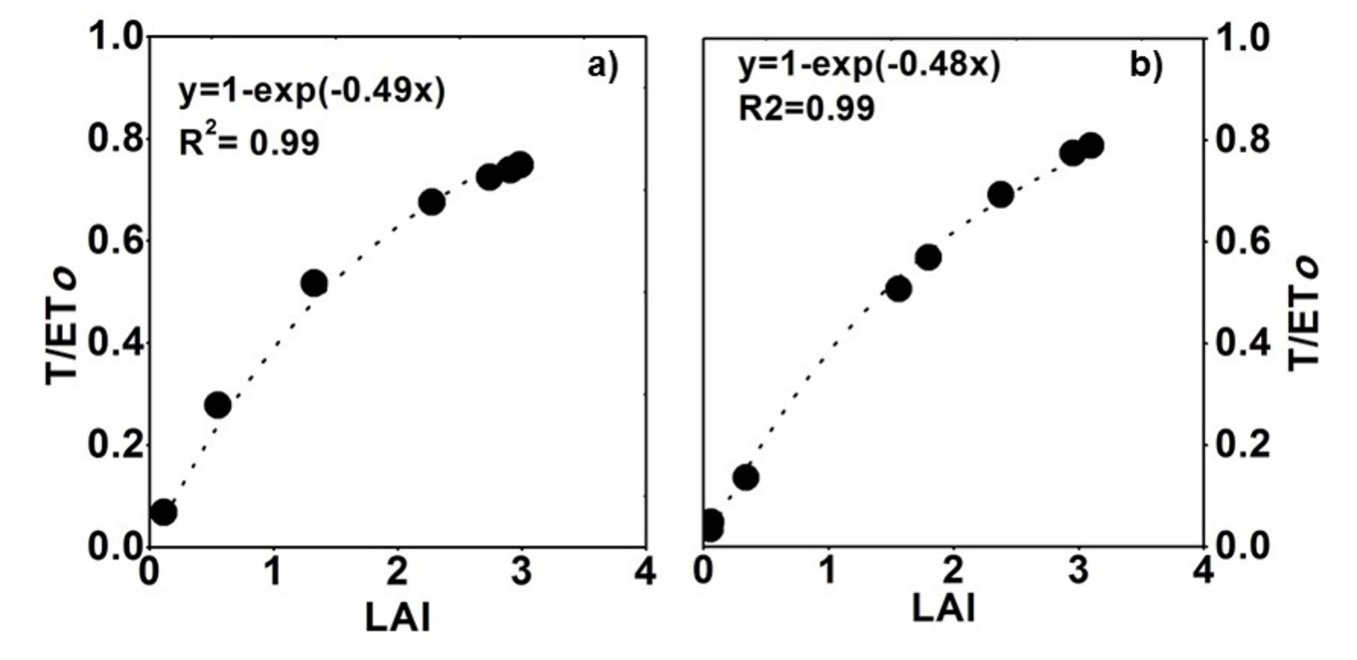

Supplement: S1 Fig — Relationship between T/ETo and LAI of (a) paddy and (b) rainfed rice.LAI was calculated as leaf area per ground area where Leaf area (LA) was determined with a Leaf Area Meter (LI−3000A, LI−COR, USA). T/ETo was calculated as the ratio of estimated daily transpiration of the LAI measurement date (Eqs 3 and 6) to estimated reference evapotranspiration (Eq 3). (PNG) [file pone.0195238.s001.png]

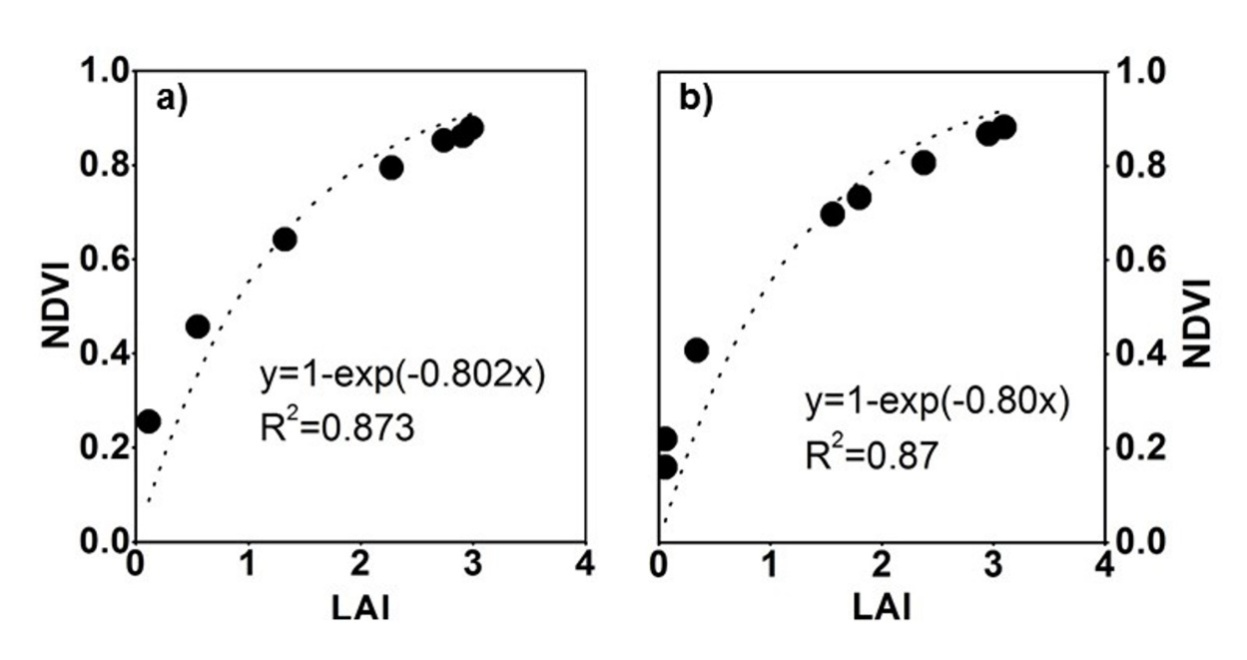

Supplement: S2 Fig — Relationship between NDVI and LAI of (a) paddy rice and (b) rainfed rice. LAI was calculated as leaf area per ground area where Leaf area (LA) was determined with a Leaf Area Meter (LI−3000A, LI−COR, USA). NDVI was measured by Cropscan (Cropscan Inc., USA). (PNG) [file pone.0195238.s002.png]

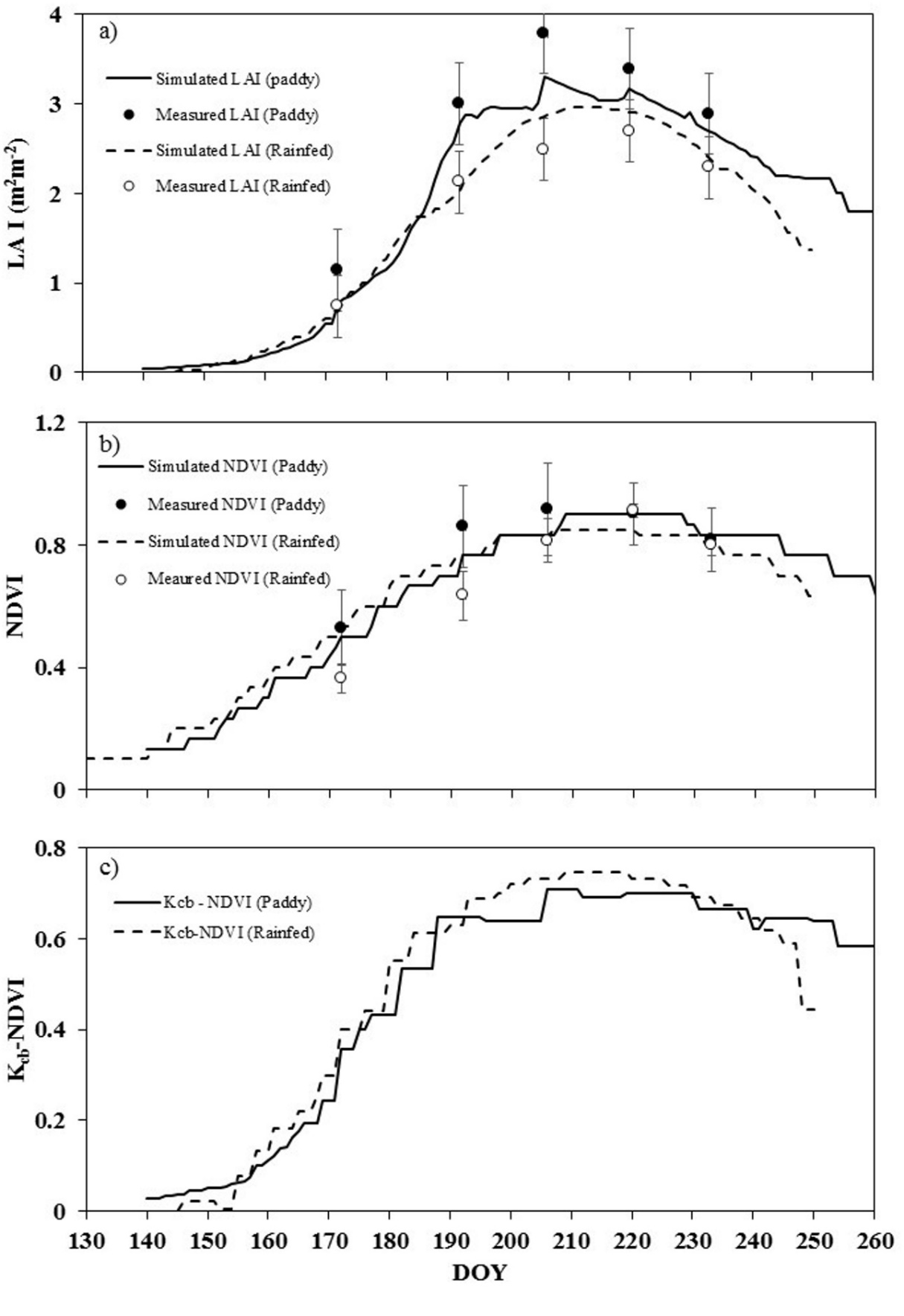

Supplement: S3 Fig — Simulated daily crop growth of paddy and rainfed rice a) LAI, b) NDVI, c) Dual crop coefficient (Kcb). Daily Kcb was simulated based on daily NDVI, after following Choudhury 1994. (PNG) [file pone.0195238.s003.png]

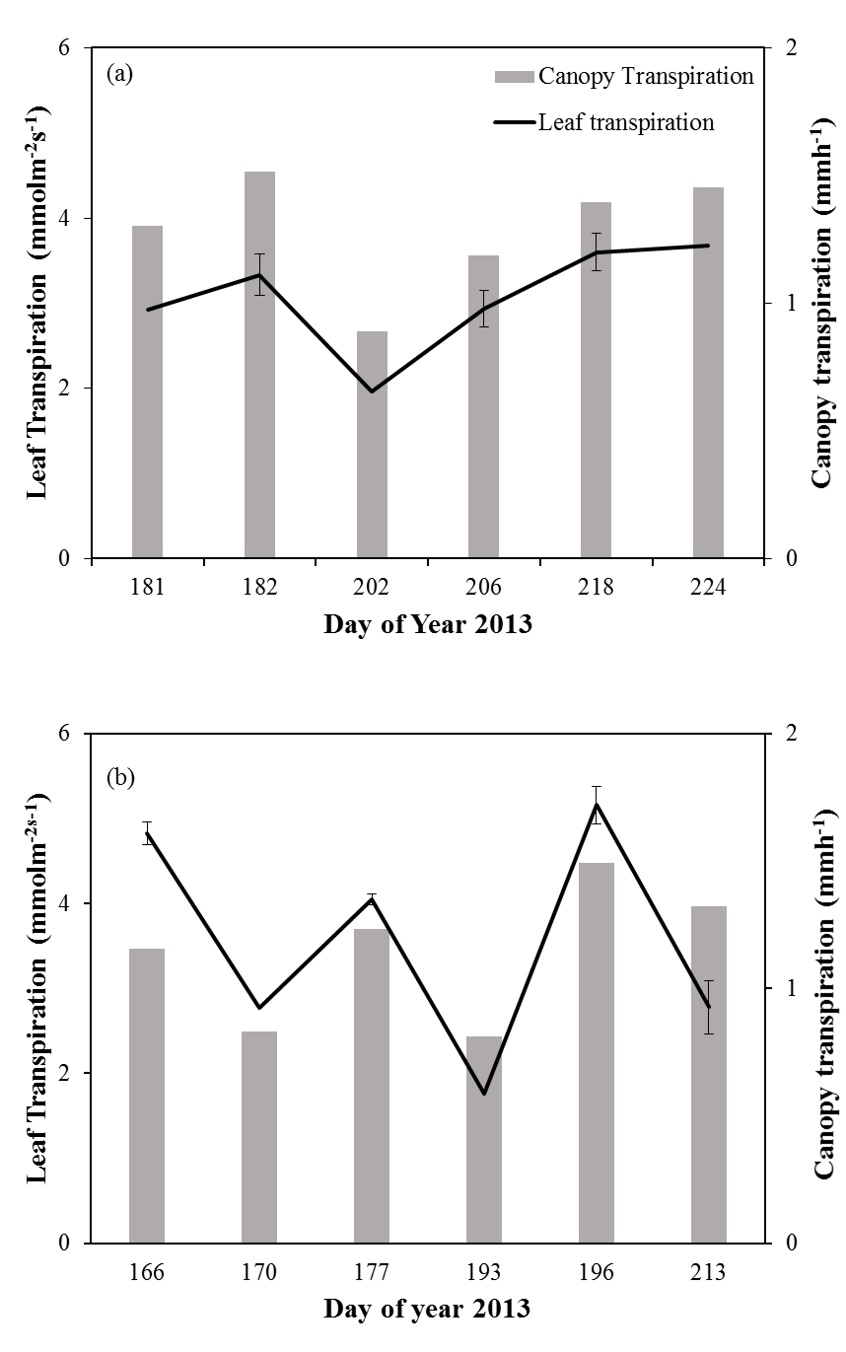

Supplement: S4 Fig — Simulated canopy transpiration followed the seasonal trends of measured leaf transpiration (Mean ± SE) of (a) rainfed rice (b) paddy rice. (PNG) [file pone.0195238.s004.png]

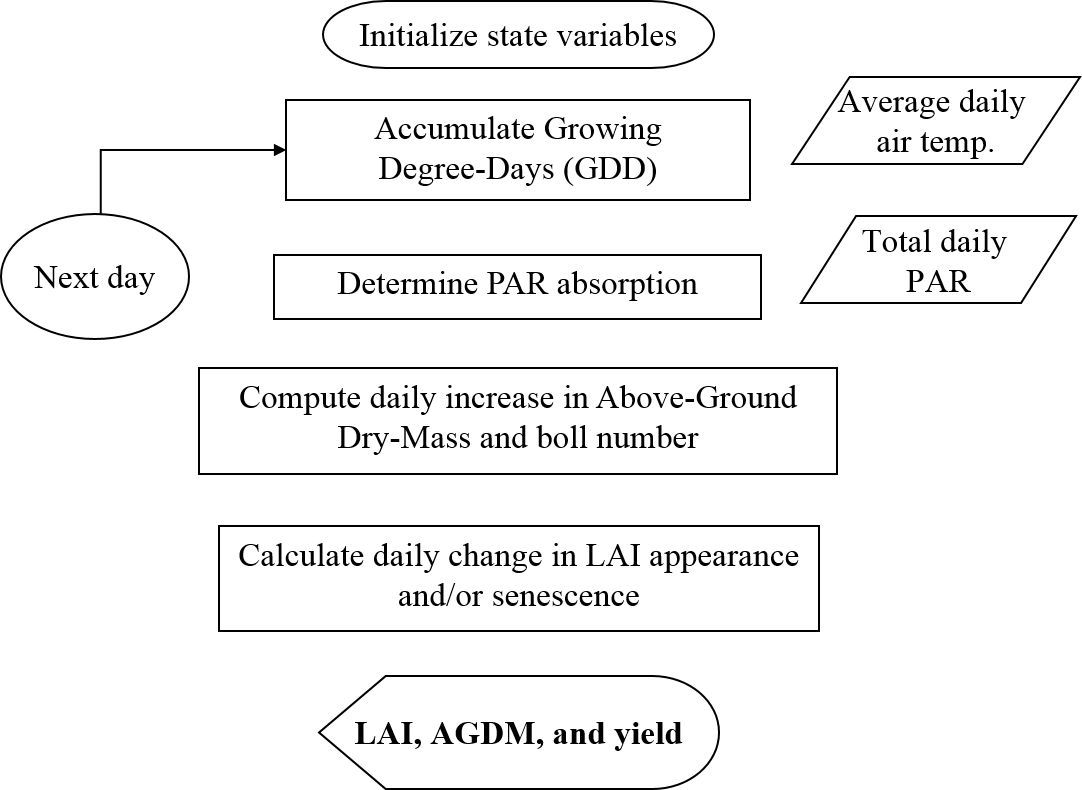

Supplement: S5 Fig — (PNG) [file pone.0195238.s005.png]
