# Supplementary material for: Quantifying differences in water and carbon cycling between paddy and rainfed rice (Oryza sativa L.) by flux partitioning
Source: PLoS One. 2018 Apr 6;13(4):e0195238. doi: 10.1371/journal.pone.0195238 (PMC5889072; doi:10.1371/journal.pone.0195238)
Supplement: S1 Table — (DOCX) [file pone.0195238.s006.docx]

**S1 Table: Field management activities and timeline in DOY**

| ***Activities*** | ***Details*** | ***Date (Show by DOY)*** | |
| --- | --- | --- | --- |
|  |  | ***Paddy*** | ***Upland*** |
| **Land Preparation** | Plowing | 130 | 107 |
|  | Harrowing | 135 | 107 |
|  | Leveling | 136 | 107 |
| **Fertilization (N:P:K= 11:5:6)** | Basal fertilization (N:P:K= 80%: 100%: 65%) | 139 | 112 |
|  | 2^nd^ dressing (N:P:K= 20%: 0%: 35%) | 158 | 160 |
| **Sowing / Transplanting** | Transplanting / Sowing | 140 | 112 |
| **Pest and Disease Control** | Disinfections for soil borne disease and pests | 140 | 106 |
| **Weed Control** | 1^st^ Herbicides application | 136 | 126 |
|  | 2^nd^ Herbicides application | X | 145 |
|  | Manual Weeding | X | 142 |
|  |  | X | 171 |
| **Water Management** | 1^st^ Irrigation (during land preparation) | 134 | X |
|  | 2^nd^ Irrigation (Permanent Flood) | 151 | X |
| **Harvest** | Harvest | 253 | 250 |
