# Supplementary material for: Quantifying differences in water and carbon cycling between paddy and rainfed rice (Oryza sativa L.) by flux partitioning
Source: PLoS One. 2018 Apr 6;13(4):e0195238. doi: 10.1371/journal.pone.0195238 (PMC5889072; doi:10.1371/journal.pone.0195238)
Supplement: S2 Table — Net Ecosystem Exchange (NEE, -NEE = GPP+ Reco) is the balance between photosynthetic uptake and release of carbon dioxide by autotrophic and heterotrophic respiration. Gross primary production (GPP) is photosynthetic uptake. Ecosystem respiration (Reco) is respiration from soil and plant. Ecosystem water use efficiency was calculated as the ratio of NEE to evapotranspiration (ET); the ratio of NEE to transpiration (T) and the ratio of GPP to T. Agronomic water use efficiency was calculated as the ratio of grain yield to ET. Differences between paddy and rainfed were tested by one way ANOVA: Carbon, water fluxes, Grain yield and water use efficiency were compared not only as crop seasonal sum but also as growth stage specific. (DOCX) [file pone.0195238.s007.docx]

**S2 Table: Water and carbon fluxes, grain yield and water use efficiency of paddy and rainfed rice**. Net Ecosystem Exchange (NEE, -NEE= GPP+ R*_eco_*) is the balance between photosynthetic uptake and release of carbon dioxide by autotrophic and heterotrophic respiration. Gross primary production (GPP) is photosynthetic uptake. Ecosystem respiration (R*_eco_*) is respiration from soil and plant. Ecosystem water use efficiency was calculated as the ratio of NEE to evapotranspiration (ET); the ratio of NEE to transpiration (T) and the ratio of GPP to T. Agronomic water use efficiency was calculated as the ratio of grain yield to ET. Differences between paddy and rainfed were tested by one way ANOVA: Carbon, water fluxes, Grain yield and water use efficiency were compared not only as crop seasonal sum but also as growth stage specific.

| **Crop** | **DOY** | **GS** | **Measured daily carbon and water fluxes** | | | | | **Grain yield (kgha^-1^)** | **Water Use Efficiency** | | | | | |
| --- | --- | --- | --- | --- | --- | --- | --- | --- | --- | --- | --- | --- | --- | --- |
|  |  |  | **GPP (gCm^-2^d^-1^)** | **NEE (gCm^-2^d^-1^)** | **R*_eco_* (gCm^-2^d^-1^)** | **ET (mmm^-2^d^-1^)** | **T (mmm^-2^d^-1^)** |  | ***NEE/ET*** | ***NEE/T*** | ***GPP/ET*** | ***GPP/T*** | **WUE *agro*** | |
|  |  |  | *(n=3)* | *(n=3)* | *(n=3)* | *(n=3)* | *(n=3)* | *(n=6)* | *(n=3)* | *(n=3)* | *(n=3)* | *(n=3)* | *(n=6)* | |
| R | 172 | S | 3.96 ± 0.27 | -1.57 ± 0.13 * | 2.35 ± 1.5 * | 1.23± 0.26 ** | 0.89 ± 0.11 | *n/a* | 1.28 ± 0.19 | 1.76 ± 0.15 * | 3.22 ± 0.51 * | 4.45 ± 0.21 | *n/a* | |
| R | 182 | T | 12.02 ± 1.53 | -3.86 ± 0.81 * | 6.01 ± 0.73 * | 1.74 ± 0.01 ** | 1.38 ± 0.08 | *n/a* | 2.22 ± 1.38 | 2.80 ± 1.12 ** | 6.91 ± 0.87 ** | 8.71 ± 0.37 | *n/a* | |
| R | 206 | H | 9.45± 0.70 ** | -1.99 ± 0.21 * | 2.19 ± 0.08 * | 0.96 ± 0.04 ** | 0.87 ± 0.01 | *n/a* | 2.08 ± 0.87 | 2.28 ± 0.66 ** | 9.84 ± 0.24 ** | 10.86 ± 0.42 | *n/a* | |
| R | 218 | M | 8.87 ± 0.70 | -2.24 ± 0.48 * | 5.74 ± 0.39 * | 1.39 ± 0.12 ** | 1.37 ± 0.01 | *n/a* | 1.61 ± 1.37 * | 1.64 ± 0.87 * | 6.38 ± 0.80 | 6.47 ± 0.47 | *n/a* | |
| P | 167 | S | 4.12 ± 0.18 | -2.92 ± 0.13 * | 1.20 ± 0.06 * | 2.27 ± 0.21 ** | 0.79 ± 0.09 | *n/a* | 1.26 ± 0.06 | 3.70 ± 0.17 ** | 1.81± 0.08 * | 5.15 ± 0.06 | *n/a* | |
| P | 175 | T | 8.68 ± 0.51 | -7.08 ± 0.42 * | 1.59 ± 0.11 * | 3.61 ± 0.38 ** | 1.11 ± 0.16 | *n/a* | 1.96 ± 0.12 | 6.38 ± 0.22 ** | 2.41 ± 0.14 ** | 7.82 ± 0.11 | *n/a* | |
| P | 200 | H | 13.19 ± 1.12 ** | -9.31 ± 0.98 * | 3.88 ± 0.20 * | 3.99 ± 0.51 ** | 1.46 ± 0.24 | *n/a* | 2.33 ± 0.25 | 6.38 ± 0.17 ** | 3.29± 0.28 ** | 9.01 ± 0.28 | *n/a* | |
| P | 219 | M | 10.84 ± 0.03 | -6.65 ± 0.27 * | 4.19 ± 0.26 * | 1.66 ± 0.28 ** | 1.55 ± 0.18 | *n/a* | 4.01 ± 0.16 * | 4.29 ± 0.11 ** | 6.53± 0.02 | 6.99 ± 0.02 | *n/a* | |
| R | crop season total | | 779.08 ±265.43 | *399.25 ±147.32 *** | *379.53±242.48 *** | 138.75 ±68.43 ** | 89.79 ±57.35 | 5989 ±683 | 2.88 ±0.22 | 4.44 ± 0.63 ** | 5.61 ± 0.94 * | 8.68 ± 1.87 | 5.58 ± 0.79 ** | |
| P | crop season total | | 832.43 ±189.78 | *552.13 ±211.54 *** | *281.30±161.32 *** | 239.88 ±87.45 ** | 100.91 ±34.32 | 6612 ±218 | 2.30 ±0.41 | 5.47 ± 0.17 ** | 3.47 ± 0.19 * | 8.25 ± 0.48 | 3.68 ± 0.21 ** |  |

* one way ANOVA between RF-PD followed by TukeyHSD test (significant at p < 0.05)

** one way ANOVA between RF-PD followed by TukeyHSD test (significant at p < 0.01)

Note: GS= growth stages, R= Rainfed, P= Paddy, S= seedling, T= Tillering, H = Heading, M= Maturity. Numbers followed the ± sign is SD.
