# Supplementary material for: Quantifying differences in water and carbon cycling between paddy and rainfed rice (Oryza sativa L.) by flux partitioning
Source: PLoS One. 2018 Apr 6;13(4):e0195238. doi: 10.1371/journal.pone.0195238 (PMC5889072; doi:10.1371/journal.pone.0195238)
Supplement: S3 Table — (DOCX) [file pone.0195238.s008.docx]

**S3 Table: Correlation matrix of carbon and water fluxes and environmental variables of paddy rice**

|  | **GPP** | **NEE** | **Reco** | **ET** | **T** | **Radiation** | **Tair** | **Tsoil** | **VPD** | **SWC** | **Windspeed** |
| --- | --- | --- | --- | --- | --- | --- | --- | --- | --- | --- | --- |
| **GPP** |  |  |  |  |  |  |  |  |  |  |  |
| Spearman's (* ρ* ) |  | -0.96 | 0.57 | 0.46 | 0.84 | 0.52 | 0.57 | 0.71 | 0.43 | -0.41 | 0.46 |
| P |  | 0.00 | 0.00 | 0.00 | 0.00 | 0.00 | 0.00 | 0.00 | 0.00 | 0.00 | 0.00 |
| **NEE** |  |  |  |  |  |  |  |  |  |  |  |
| Spearman's (* ρ* ) | -0.96 |  | -0.37 | -0.60 | -0.76 | -0.62 | -0.39 | -0.59 | -0.47 | 0.26 | -0.39 |
| P | 0.00 |  | 0.00 | 0.00 | 0.00 | 0.00 | 0.00 | 0.00 | 0.00 | 0.00 | 0.00 |
| **Reco** |  |  |  |  |  |  |  |  |  |  |  |
| Spearman's (* ρ* ) | 0.57 | -0.37 |  | -0.15 | 0.70 | 0.04 | 0.90 | 0.78 | 0.20 | -0.80 | 0.42 |
| P | 0.00 | 0.00 |  | 0.11 | 0.00 | 0.67 | 0.00 | 0.00 | 0.03 | 0.00 | 0.00 |
| **ET** |  |  |  |  |  |  |  |  |  |  |  |
| Spearman's (* ρ* ) | 0.46 | -0.60 | -0.15 |  | 0.36 | 0.87 | 0.00 | 0.26 | 0.67 | 0.07 | 0.20 |
| P | 0.00 | 0.00 | 0.11 |  | 0.00 | 0.00 | 0.96 | 0.00 | 0.00 | 0.45 | 0.03 |
| **T** |  |  |  |  |  |  |  |  |  |  |  |
| Spearman's (* ρ* ) | 0.84 | -0.76 | 0.70 | 0.36 |  | 0.48 | 0.63 | 0.70 | 0.44 | -0.62 | 0.25 |
| P | 0.00 | 0.00 | 0.00 | 0.00 |  | 0.00 | 0.00 | 0.00 | 0.00 | 0.00 | 0.01 |
| **Radiation** |  |  |  |  |  |  |  |  |  |  |  |
| Spearman's (* ρ* ) | 0.52 | -0.62 | 0.04 | 0.87 | 0.48 |  | 0.06 | 0.30 | 0.78 | -0.18 | 0.24 |
| P | 0.00 | 0.00 | 0.67 | 0.00 | 0.00 |  | 0.51 | 0.00 | 0.00 | 0.05 | 0.01 |
| **Tair** |  |  |  |  |  |  |  |  |  |  |  |
| Spearman's (* ρ* ) | 0.57 | -0.39 | 0.90 | 0.00 | 0.63 | 0.06 |  | 0.86 | 0.29 | -0.70 | 0.55 |
| P | 0.00 | 0.00 | 0.00 | 0.96 | 0.00 | 0.51 |  | 0.00 | 0.00 | 0.00 | 0.00 |
| **Tsoil** |  |  |  |  |  |  |  |  |  |  |  |
| Spearman's (* ρ* ) | 0.71 | -0.59 | 0.78 | 0.26 | 0.70 | 0.30 | 0.86 |  | 0.38 | -0.67 | 0.48 |
| P | 0.00 | 0.00 | 0.00 | 0.00 | 0.00 | 0.00 | 0.00 |  | 0.00 | 0.00 | 0.00 |
| **VPD** |  |  |  |  |  |  |  |  |  |  |  |
| Spearman's (* ρ* ) | 0.43 | -0.47 | 0.20 | 0.67 | 0.44 | 0.78 | 0.29 | 0.38 |  | -0.34 | 0.39 |
| P | 0.00 | 0.00 | 0.03 | 0.00 | 0.00 | 0.00 | 0.00 | 0.00 |  | 0.00 | 0.00 |
| **SWC** |  |  |  |  |  |  |  |  |  |  |  |
| Spearman's (* ρ* ) | -0.41 | 0.26 | -0.80 | 0.07 | -0.62 | -0.18 | -0.70 | -0.67 | -0.34 |  | -0.17 |
| P | 0.00 | 0.00 | 0.00 | 0.45 | 0.00 | 0.05 | 0.00 | 0.00 | 0.00 |  | 0.06 |
| **Windspeed** |  |  |  |  |  |  |  |  |  |  |  |
| Spearman's (* ρ* ) | 0.46 | -0.39 | 0.42 | 0.20 | 0.25 | 0.24 | 0.55 | 0.48 | 0.39 | -0.17 |  |
| P | 0.00 | 0.00 | 0.00 | 0.03 | 0.01 | 0.01 | 0.00 | 0.00 | 0.00 | 0.06 |  |
