# Supplementary material for: Quantifying differences in water and carbon cycling between paddy and rainfed rice (Oryza sativa L.) by flux partitioning
Source: PLoS One. 2018 Apr 6;13(4):e0195238. doi: 10.1371/journal.pone.0195238 (PMC5889072; doi:10.1371/journal.pone.0195238)
Supplement: S4 Table — (DOCX) [file pone.0195238.s009.docx]

**S4 Table: Correlation matrix of carbon and water fluxes and environmental variables of rainfed rice**

|  | **GPP** | **NEE** | **Reco** | **ET** | **T** | **Radiation** | **Tair** | **Tsoil** | **VPD** | **SWC** | **Windspeed** |
| --- | --- | --- | --- | --- | --- | --- | --- | --- | --- | --- | --- |
| **GPP** |  |  |  |  |  |  |  |  |  |  |  |
| Spearman's (* ρ* ) |  | -0.81 | 0.89 | 0.71 | 0.79 | 0.25 | 0.72 | 0.80 | 0.20 | -0.42 | 0.56 |
| P |  | 0.00 | 0.00 | 0.00 | 0.00 | 0.01 | 0.00 | 0.00 | 0.03 | 0.00 | 0.00 |
| **NEE** |  |  |  |  |  |  |  |  |  |  |  |
| Spearman's (* ρ* ) | -0.81 |  | -0.50 | -0.66 | -0.67 | -0.53 | -0.46 | -0.58 | -0.43 | 0.36 | -0.43 |
| P | 0.00 |  | 0.00 | 0.00 | 0.00 | 0.00 | 0.00 | 0.00 | 0.00 | 0.00 | 0.00 |
| **Reco** |  |  |  |  |  |  |  |  |  |  |  |
| Spearman's (* ρ* ) | 0.89 | -0.50 |  | 0.55 | 0.65 | -0.01 | 0.74 | 0.80 | 0.00 | -0.35 | 0.55 |
| P | 0.00 | 0.00 |  | 0.00 | 0.00 | 0.90 | 0.00 | 0.00 | 0.97 | 0.00 | 0.00 |
| **ET** |  |  |  |  |  |  |  |  |  |  |  |
| Spearman's (* ρ* ) | 0.71 | -0.66 | 0.55 |  |  | 0.43 | 0.65 | 0.57 | 0.47 | -0.36 | 0.43 |
| P | 0.00 | 0.00 | 0.00 |  |  | 0.00 | 0.00 | 0.00 | 0.00 | 0.00 | 0.00 |
| **T** |  |  |  |  |  |  |  |  |  |  |  |
| Spearman's (* ρ* ) | 0.79 | -0.67 | 0.65 | 0.89 |  | 0.23 | 0.76 | 0.59 | 0.28 | -0.56 | 0.39 |
| P | 0.00 | 0.00 | 0.00 | 0.00 |  | 0.01 | 0.00 | 0.00 | 0.00 | 0.00 | 0.00 |
| **Radiation** |  |  |  |  |  |  |  |  |  |  |  |
| Spearman's (* ρ* ) | 0.25 | -0.53 | -0.01 | 0.43 | 0.23 |  | -0.02 | 0.10 | 0.81 | -0.04 | 0.12 |
| P | 0.01 | 0.00 | 0.90 | 0.00 | 0.01 |  | 0.86 | 0.27 | 0.00 | 0.65 | 0.21 |
| **Tair** |  |  |  |  |  |  |  |  |  |  |  |
| Spearman's (* ρ* ) | 0.72 | -0.46 | 0.74 | 0.65 | 0.76 | -0.02 |  | 0.82 | 0.19 | -0.58 | 0.62 |
| P | 0.00 | 0.00 | 0.00 | 0.00 | 0.00 | 0.86 |  | 0.00 | 0.03 | 0.00 | 0.00 |
| **Tsoil** |  |  |  |  |  |  |  |  |  |  |  |
| Spearman's (* ρ* ) | 0.80 | -0.58 | 0.80 | 0.57 | 0.59 | 0.10 | 0.82 |  | 0.22 | -0.38 | 0.72 |
| P | 0.00 | 0.00 | 0.00 | 0.00 | 0.00 | 0.27 | 0.00 |  | 0.01 | 0.00 | 0.00 |
| **VPD** |  |  |  |  |  |  |  |  |  |  |  |
| Spearman's (* ρ* ) | 0.20 | -0.43 | 0.00 | 0.47 | 0.28 | 0.81 | 0.19 | 0.22 |  | -0.25 | 0.24 |
| P | 0.03 | 0.00 | 0.97 | 0.00 | 0.00 | 0.00 | 0.03 | 0.01 |  | 0.01 | 0.01 |
| **SWC** |  |  |  |  |  |  |  |  |  |  |  |
| Spearman's (* ρ* ) | -0.42 | 0.36 | -0.35 | -0.36 | -0.56 | -0.04 | -0.58 | -0.38 | -0.25 |  | -0.26 |
| P | 0.00 | 0.00 | 0.00 | 0.00 | 0.00 | 0.65 | 0.00 | 0.00 | 0.01 |  | 0.00 |
| **Windspeed** |  |  |  |  |  |  |  |  |  |  |  |
| Spearman's (* ρ* ) | 0.56 | -0.43 | 0.55 | 0.43 | 0.39 | 0.12 | 0.62 | 0.72 | 0.24 | -0.26 |  |
| P | 0.00 | 0.00 | 0.00 | 0.00 | 0.00 | 0.21 | 0.00 | 0.00 | 0.01 | 0.00 |  |
