# Supplementary material for: Quantifying differences in water and carbon cycling between paddy and rainfed rice (Oryza sativa L.) by flux partitioning
Source: PLoS One. 2018 Apr 6;13(4):e0195238. doi: 10.1371/journal.pone.0195238 (PMC5889072; doi:10.1371/journal.pone.0195238)
Supplement: S5 Table — Mk, PT, 56PM, m56PM80, m56PM100, m56PM120 are conventional reference crop ET (ET0, grass as reference crop) estimation methods while m56PMmrc is reference crop ET of rice (ET0, healthy and well-watered rice as reference crop). Kcb_FAO is the FAO recommended hypothetical basal crop coefficients (Provided in section (4.2.1), Table (4.2) while Kcb_NDVI is NDVI derived basal crop coefficient. R2 is determination of coefficients, RMSE is root mean square error, p (t-test) is level of significant of the test, CV (RMSE) is coefficient of variation determined by RMSE, ME (Nseff) is model efficiency and Score is the score of model performance ranked based on ME and R2. (DOCX) [file pone.0195238.s010.docx]

**S5 Table:** Comparison of different crop ET estimation methods. Mk, PT, 56PM, m56PM_80_, m56PM_100_, m56PM_120_ are conventional reference crop ET (ET_0_, grass as reference crop) estimation methods while m56PM_mrc_ is reference crop ET of rice (ET_0_, healthy and well-watered rice as reference crop). K_cb_FAO_ is the FAO recommended hypothetical basal crop coefficients (Provided in section (4.2.1), Table (4.2) while K_cb_NDVI_ is NDVI derived basal crop coefficient. R^2^ is determination of coefficients, RMSE is root mean square error, p (t-test) is level of significant of the test, CV (RMSE) is coefficient of variation determined by RMSE, ME (Nseff) is model efficiency and Score is the score of model performance ranked based on ME and R^2^.

| **Crop ET estimates** | **R^2^** | ***p* (t-test)** | | **RMSE** | | **CV (RMSE)** | | **ME (Nseff)** | | **Score** | |
| --- | --- | --- | --- | --- | --- | --- | --- | --- | --- | --- | --- |
|  | n=6 | | | | | | | | | |  |
| *Mk + K_cb_FAO_* | 0.70 | <0.05 | 0.21 | | 0.17 | | -1.76 | | 6 | |  |
| *Mk + K_cb_NDVI_* | 0.47 | 0.13 | 0.28 | | 0.23 | | -5.74 | | 8 | |  |
| *PT +K_cb_FAO_* | 0.43 | 0.16 | 0.30 | | 0.24 | | -10.53 | | 9 | |  |
| *PT +K_cb_NDVI_* | 0.05 | 0.68 | 0.38 | | 0.31 | | -30.80 | | 10 | |  |
| *56PM + K_cb_FAO_* | 0.64 | 0.06 | 0.24 | | 0.19 | | 0.52 | | 2 | |  |
| *56PM + K_cb_NDVI_* | 0.55 | <0.05 | 0.26 | | 0.21 | | -0.05 | | 3 | |  |
| *m56PM_80+_ K_cb_FAO_* | 0.45 | 0.14 | 0.29 | | 0.23 | | -0.68 | | 5 | |  |
| *m56PM_80+_ K_cb_NDVI_* | 0.21 | 0.36 | 0.35 | | 0.28 | | -3.35 | | 7 | |  |
| *m56PM_100+_ K_cb_FAO_* | 0.46 | 0.14 | 0.29 | | 0.23 | | -0.33 | | 4 | |  |
| *m56PM_100+_ K_cb_NDVI_* | 0.21 | 0.35 | 0.35 | | 0.28 | | -2.59 | | 7 | |  |
| *m56PM_120+_ K_cb_FAO_* | 0.45 | 0.08 | 0.29 | | 0.23 | | 0.52 | | 2 | |  |
| *m56PM_120+_ K_cb_NDVI_* | 0.45 | <0.05 | 0.25 | | 0.20 | | 0.57 | | 2 | |  |
| *m56PM_mrc_+ K_cb_FAO_* | 0.75 | <0.05 | 0.20 | | 0.16 | | 0.50 | | 2 | |  |
| *m56PM_mrc_+ K_cb_NDVI_* | 0.94 | <0.05 | 0.10 | | 0.08 | | 0.76 | | 1 | |  |
