# Supplementary material for: Quantifying differences in water and carbon cycling between paddy and rainfed rice (Oryza sativa L.) by flux partitioning
Source: PLoS One. 2018 Apr 6;13(4):e0195238. doi: 10.1371/journal.pone.0195238 (PMC5889072; doi:10.1371/journal.pone.0195238)
Supplement: S1 File — (DOCX) [file pone.0195238.s011.docx]

**Supporting file 1**

**Calculation of Net Radiation**

Net radiation was calculated based on solar radiation as suggested by Allen et al. (1998):

(1) Calculation of net short wave radiation from solar radiation

Rns = (1-0.23)*Rs (Monteith and Szeicz, 1960);

where Rns= net short wave radiation, 0.23 is an estimated albedo of crop cover and Rs is solar radiation.

(2) Calculation of net long wave radiation according to Stefan-Boltzmann Law

Rnl=σ [(Tmax+Tmin)/2]*(0.34-(0.14*√e0)*[1.35*(Rs/Rso)-0.35]

Where, Rnl is net long wave radiation, s is Stephan-Boltzmann constant, 4.903*10^-9^ MJK^-4^m^-2^day^-1^ and Rs is solar radiation, which is measured and Rso is clear sky radiation calculated as follow:

Rso=(0.75+(210^-5^*z)*Rs

Where, Rso is clear sky radiation, z is elevation above sea level (m), Rs is solar radiation, 0.75 and 210 calibrated values recommended by FAO 56 PM model of Allen et al., 1998.

**ET estimation by FAO 56 dual crop approach with NDVI K_cb_ method against other ET estimation methods**

Radiation driven (Makkink (1957) (*Mk*) and Priestley and Taylor (1972) (*PT*) models) and Penman - Monteith (1965) type combination methods (the 56PM model and modifications) of ET estimation methods were tested against the chamber measured ET (Table S4). The FAO 56 dual crop with *NDVI K_cb_* model, which used the measured growth stage average leaf resistance of rice in combination with *NDVI* derived basal crop coefficient (*m56PM_mrc_+K_cb_NDVI_*) performed the best (R^2^=0.95, p< 0.05, RMSE = 0.10, NSeff ME = 0.76, CV (RMSE) = 0.08). The original 56PM (*56PM* with canopy conductance of 70 sm^-1^as described in Allen et al., 1998) model in combination with both FAO recommended *K_cb_* values and NDVI derived *K_cb_* values performed better than PT and Mk (Table S4). However, applying fixed canopy conductance 80, 100 and 120 sm^-1^ (*m56PM_80_, m56PM_100_, m56PM_120_*) instead of the FAO 56 recommended 70 sm^-1^ showed better model performance with higher modelling efficiency. Among the compared different ET models; two radiation based models and one Penman type combination model, the *PM* type models performed better than radiation based models of the Makkink 1957 and the Priestley-Taylor 1972. However, in the case of limited meteorological data availability to perform FAO 56 dual crop model, radiation based Makkink, 1957 (*Mk*) in combination with the FAO 56 recommended *K_cb_* values would be an option to estimate crop evapotranspiration as it performed better (R^2^ = 0.70, p < 0.05, RMSE = 0.21, ME (Nseff) = -1.76, CV (RMSE) = 0.17) than another radiation based model, Priestley-Taylor (the *PT*, 1972). In this study, we used the .FAO 56 dual crop approach with NDVI derived Kcb (*m56PM_mrc_+K_cb_NDVI_*).

**The seasonal trends of measured leaf transpiration and simulated canopy transpiration**

Leaf transpiration (at controlled leaf cuvette microenvironment at CO_2_ concentration of 400 μmol mol^-1^ and PAR of 1500 μmol m^-2^ s^-1^) of the fully developed uppermost rice leaf (*n=3-5*) was measured by a portable gas exchange analyzer (GFS-3000, Heinz Walz GmbH, Effeltrich, Germany), to see the seasonal transpiration trends of both paddy and rainfed rice. Simulated daily canopy transpiration followed the measured leaf transpiration trends (Figure S4).
